# Conformational reprogramming of c-Myc *via* intracellular cyclisation

**DOI:** 10.1039/d6cb00191b

**Published:** 2026-09-15

**Authors:** Rachel A. Johnson, Jody M. Mason

**Affiliations:** a Department of Life Sciences, University of Bath Bath BA2 7AY UK j.mason@bath.ac.uk; b Revolver Therapeutics, Innovation Centre, University of Bath BA2 7AY UK

## Abstract

Intrinsically disordered transcription factors represent a major challenge for chemical biology. Their lack of persistent structure limits both functional interrogation and ligand discovery. Here, we show that intracellular covalent cyclisation can conformationally reprogram the intrinsically disordered c-Myc bHLHZip region into a more ordered, DNA-binding state independent of its obligate partner Max. Using cell-penetrating bis-alkylating crosslinkers and rationally positioned cysteine pairs, we introduced helix-stabilising constraints into c-Myc during intracellular recombinant expression in *E. coli*. Among multiple designs, a single constraint positioned within helix two uniquely induced helicity and increased thermal stability, enabling sequence-specific E-box binding in the absence of Max. Notably, this gain of function does not recapitulate the canonical Myc-Max coiled coil architecture, revealing that non-native but functional conformational states can support sequence-specific DNA recognition. These findings demonstrate that intracellular chemical constraint can provide access to structured states that are normally inaccessible in the unbound protein and obscured within native protein complexes. By decoupling conformational ordering from partner occupancy, this approach establishes conformational reprogramming as a strategy to render otherwise intractable proteins amenable to functional interrogation and ligand discovery.

## Abstract 2

The c-Myc bHLHZip can be covalently modified through dithiol bis-alkylation during recombinant expression. Stapling in helix two induces helicity and enables Max-independent E-box binding while retaining Myc-Max complex formation.

## Graphical abstract

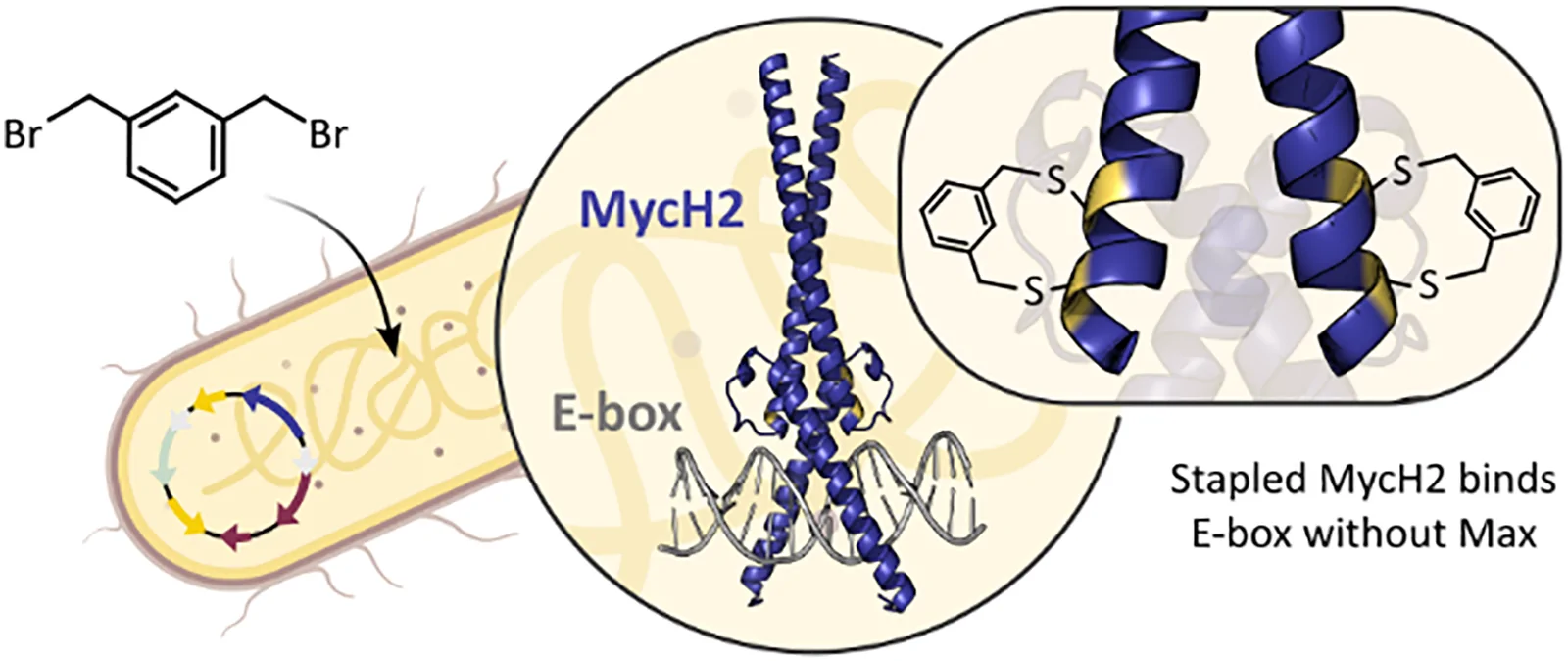

## Introduction

Intrinsically disordered proteins (IDPs) represent a major frontier for chemical biology. Their dynamic conformational landscapes, lack of persistent secondary structure, and frequent reliance on binding partners for functional ordering have rendered many IDPs refractory to traditional small-molecule and biologic approaches.^1^ Transcription factors are particularly prominent within this class, as their regulatory activity often emerges only upon partner-induced folding and assembly on DNA. This creates a central challenge for ligand discovery: structured and potentially targetable surfaces may exist only transiently, or only within partner-bound states where those same surfaces are remodelled or obscured. Developing chemical strategies that can directly manipulate the conformational states of IDPs inside cells therefore represents a fundamental challenge and opportunity for the field.

The oncogenic transcription factor c-Myc exemplifies this problem. It is thought to regulate as much as 15% of the human genome and is often dysregulated in a wide range of cancers.^2–4^ In isolation, c-Myc is intrinsically disordered and incapable of stable DNA binding, acquiring α-helical structure only upon heterodimerisation with its obligate partner Max through the basic helix-loop-helix leucine zipper (bHLHZip) domain (Fig. 1A).^5–7^ This partner dependence has dominated both mechanistic understanding and therapeutic strategy. Peptide and protein-based approaches typically aim to disrupt the Myc-Max interaction, mimic Max to sequester Myc in inactive complexes or sterically block the Myc-Max heterodimer from binding E-box DNA.^8–14^ Despite extensive effort, these strategies have not addressed a more fundamental question: whether the conformational and functional limitations of c-Myc are immutable properties of the protein, or whether they can be chemically modulated. More broadly, c-Myc illustrates a paradox common to many IDPs: the cognate binding partner that induces structure can also occupy the same surface that one may wish to interrogate or target. A strategy that imposes structure without requiring native partner engagement could therefore reveal cryptic conformational states that are folded, functional and more amenable to ligand discovery.

**Fig. 1:**
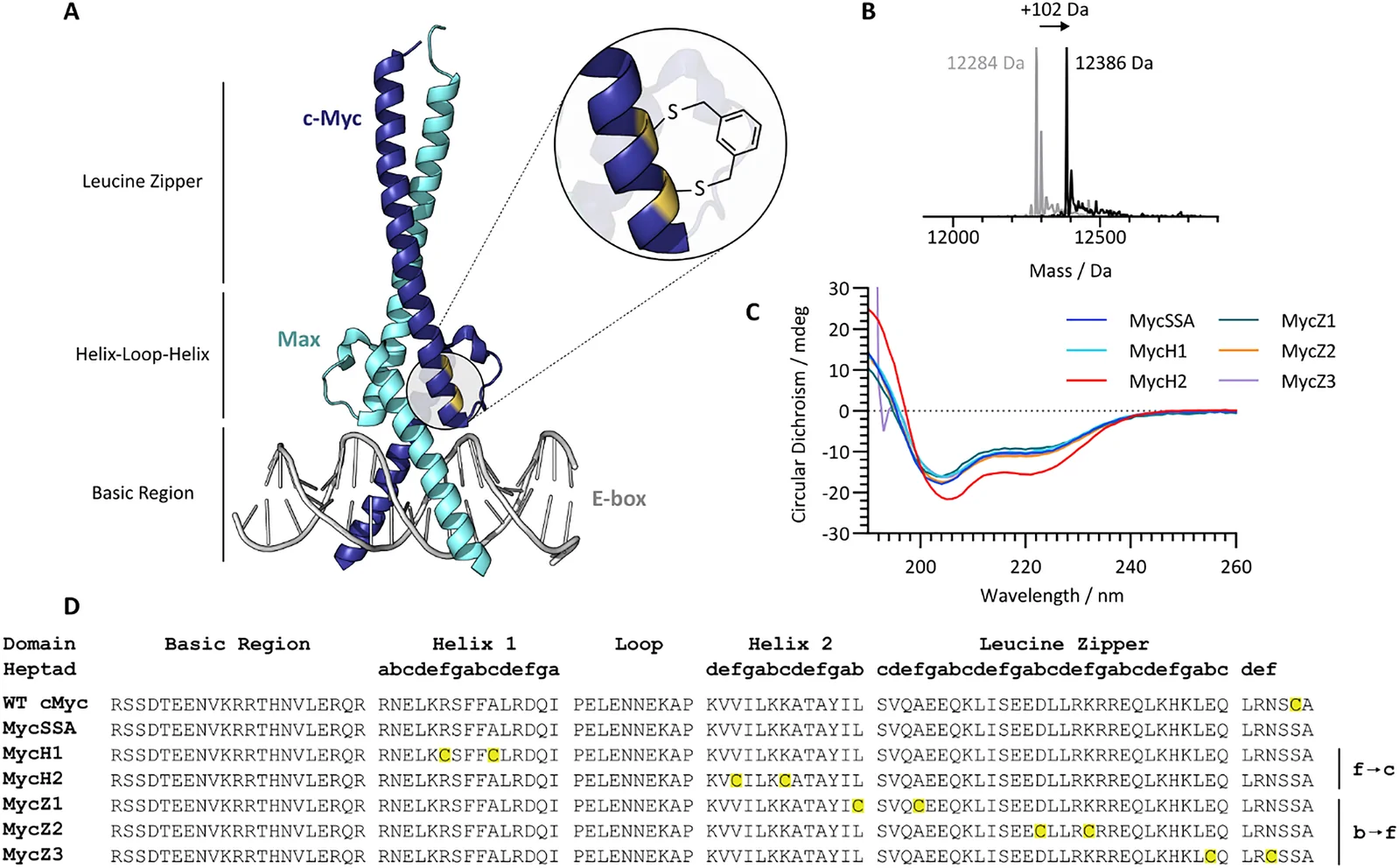
Site-specific chemical reprogramming of c-Myc during intracellular recombinant expression. (A) Schematic representation of the c-Myc bHLHZip region highlighting the introduction of a mDBMB-derived chemical crosslinker in helix two. (PDB: 1NKP)^7^ (B) Representative mass spectrometry analysis of a stapled c-Myc construct showing the expected +102 Da mass increase consistent with intramolecular cyclisation. (C) Circular dichroism analysis of all c-Myc constructs analysed alone, with MycH2 observed as the sole construct to exhibit an increase in helicity as a result of stapling. (D) Sequences of all c-Myc constructs showing the position of the cysteine pairs spanning the helix-loop-helix and leucine zipper of c-Myc. MycH1 and MycH2 target f → c positions in helix one and helix two, respectively, whereas MycZ1–3 target b → f positions within the leucine zipper.

We address this question by chemically reprogramming c-Myc through the introduction of intracellular covalent constraints during protein production. The application of chemical crosslinkers to direct peptide topology is well established, particularly the use of *i*, *i* + 4 and *i*, *i* + 7 side chain-to-side chain modification to induce and stabilise α-helical secondary structure.^15,16^ We previously demonstrated that cell-penetrating bis-alkylating crosslinkers can cyclise genetically encoded peptide libraries directly within living cells.^17^ Here, we extend this concept to an aggregation-prone, intrinsically disordered c-Myc bHLHZip construct and ask whether intracellular chemical constraint can actively reprogramme its conformational and functional state by enforcing helicity within this region. In doing so, we test whether a structured and DNA-competent state of c-Myc can be induced chemically without requiring occupancy by Max.

## Results and discussion

To enable site-specific cyclisation, the native C-terminal Cys438 of c-Myc was first mutated to serine to prevent off-target alkylation. This generated an unstapled control construct, termed MycSSA to reflect the modified C-terminal sequence. Cysteine pairs were introduced at solvent-facing heptad positions to favour intramolecular crosslinking without disrupting the hydrophobic core of the coiled-coil interface. We focused on *i*, *i +* 4 spacings, which optimally reinforce α-helical geometry, and positioned cysteine substitutions at b → f or f → c heptad registers across helix one, helix two, and the leucine zipper (Fig. 1A and D). This strategy allowed us to systematically test the effect of crosslinker position on helix induction and the formation of DNA-binding conformations.

The bis-alkylating agent 1,3-bis(bromomethyl)benzene (mDBMB) was added directly to the media during recombinant expression of c-Myc in *E. coli.* This resulted in reproducible intracellular covalent cyclisation, as confirmed by the expected +102 Da mass increase following purification (Fig. 1B and Fig. S1). Despite the insoluble nature of recombinant c-Myc expression in *E. coli*, and the requirement for purification under denaturing conditions,^7,10,18^ cyclisation occurred efficiently during protein synthesis (Fig. S2–S6).

This approach differs fundamentally from post-purification stapling strategies. The use of intracellular cyclisation circumvents solubility constraints that severely limit *in vitro* modification of this scaffold (Fig. S7). Moreover, as previously demonstrated, this intracellular chemistry avoids synthetic peptide preparation, minimises purification steps, and reduces the use of organic solvents, providing a cheaper, cleaner and more efficient route to constrained proteins.^17^ Complete conversion was confirmed by mass spectrometry, therefore establishing that even insoluble recombinant IDP regions can be intracellularly constrained before isolation.

Circular dichroism was used to assess the extent of helix induction provided by stapling at each given position. A comparison of all constructs revealed that MycH2 was the only variant to exhibit a clear increase in α-helical content relative to the unstapled MycSSA (Fig. 1C and Fig. S8, S9). These data demonstrate that the apparent intrinsic disorder of c-Myc is not an immutable property but can be overridden through precisely positioned intracellular chemical constraint.

We next asked whether the chemically enforced helicity provided by stapling in helix two was sufficient to enable sequence-specific E-box DNA binding. Extensive structural evidence indicates that MycSSA does not homodimerise under physiological conditions or bind DNA in the absence of Max.^4,7^ Consistent with its established partner dependence, MycSSA did not produce a discrete E-box shift under the conditions shown in Fig. 2A, although weak band retardation and/or smearing was observed at the highest protein concentrations in some EMSA experiments (Fig. S10 and S11). As expected, both the MycSSA-Max heterodimer and the Max homodimer produced clear shifts, confirming assay sensitivity and specificity.

**Fig. 2:**
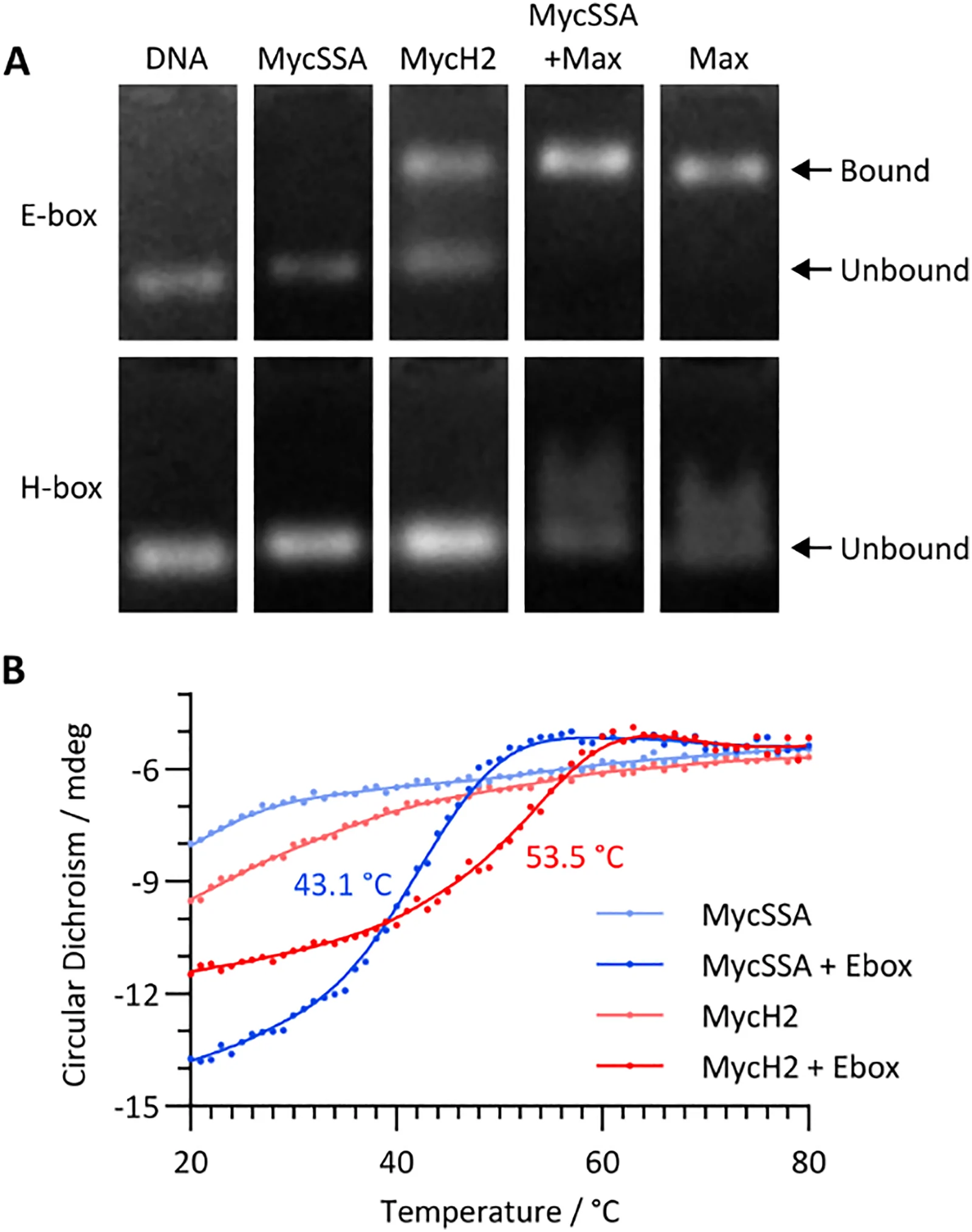
Stapling in helix two uniquely enables Max-independent, sequence-specific binding of E-box DNA. (A) Electrophoretic mobility shift assay (EMSA) demonstrating E-box DNA binding by MycH2 in the absence of Max. Unstapled MycSSA serves as a negative control; the MycSSA-Max heterodimer and Max homodimer serve as positive controls. Binding does not occur using an H-box control sequence, confirming sequence specificity. (B) Thermal denaturation of MycSSA and MycH2 alone (light blue/red), and in the presence of E-box DNA (dark blue/red). Melting temperatures were obtained by non-linear regression of normalised data (Fig. S13D).

Among all stapled constructs tested, MycH2 uniquely exhibited a clear mobility shift upon incubation with E-box DNA (Fig. 2A and Fig. S10, S11). The corresponding unstapled MycH2 cysteine mutant did not produce the same discrete E-box shift in the absence of mDBMB (Fig. S11D), indicating that the gain of Max-independent DNA-binding activity requires covalent cyclisation rather than cysteine substitution alone. An equivalent shift was also not observed using an H-box control sequence. This demonstrates that enforced helicity restores sequence-specific DNA recognition rather than non-specific nucleic acid association. However, since EMSA mobility alone does not unambiguously define stoichiometry, we therefore conservatively describe it as a Max-independent MycH2-E-box complex. These findings indicate that Max is not strictly required for E-box binding *per se*, but rather for imposing the α-helical conformation required for productive DNA engagement. Chemical crosslinking within helix two can therefore functionally substitute for the natural dimerisation partner of c-Myc.

To understand the structural basis of this gain of function, the stapled c-Myc constructs were next analysed using circular dichroism and thermal denaturation in the presence of DNA. MycH2 displayed a pronounced increase in thermal stability upon addition of E-box DNA, with a melting temperature approximately 10 °C higher than that of MycSSA (Fig. 2B and Fig. S12, S13 and Table S9). Although a thermal transition was also observed for MycSSA in the presence of E-box DNA, this was not accompanied by the discrete EMSA shift seen for MycH2 and most likely reflects weak or transient DNA-associated ordering, rather than a comparably stable Max-independent complex. Previous studies have suggested that Myc-Max heterodimerisation proceeds *via* a monomer-pathway model whereby individual protein chains interact with the DNA prior to formation of the ternary complex and this may explain the observation described above.^19,20^ Consistent with EMSA data, these transitions were strictly sequence-dependent and not observed with H-box DNA (Fig. S12). This increase in thermal stability was unique to stapling in helix two. Notably, MycH1 and MycH2 each introduce the same overall change in net charge (−1), whereas the leucine-zipper variants introduce changes of 0 to +1. The distinctive behaviour of MycH2 therefore does not correlate simply with altered net charge, but instead points to the position and local structural consequence of the imposed constraint. Stapling across the leucine zipper or in helix one had a negligible or detrimental effect on DNA binding and stability respectively (Fig. S13B).

Notably, the CD spectrum of MycH2 did not alter significantly in the presence of E-box DNA and the profile is indicative of a mixture of random coil and α-helical secondary structure. The ratio of circular dichroism at 222 nm and 208 nm can be used as a measure of coiled coil formation,^21,22^ and this remains virtually unchanged (Fig. S14 and Table S10). This indicates that though MycH2 is evidently capable of binding DNA in the absence of Max, it adopts a conformation distinct from the native, α-helical coiled coil of the Myc-Max heterodimer (Fig. 3A and Fig. S18).

**Fig. 3:**
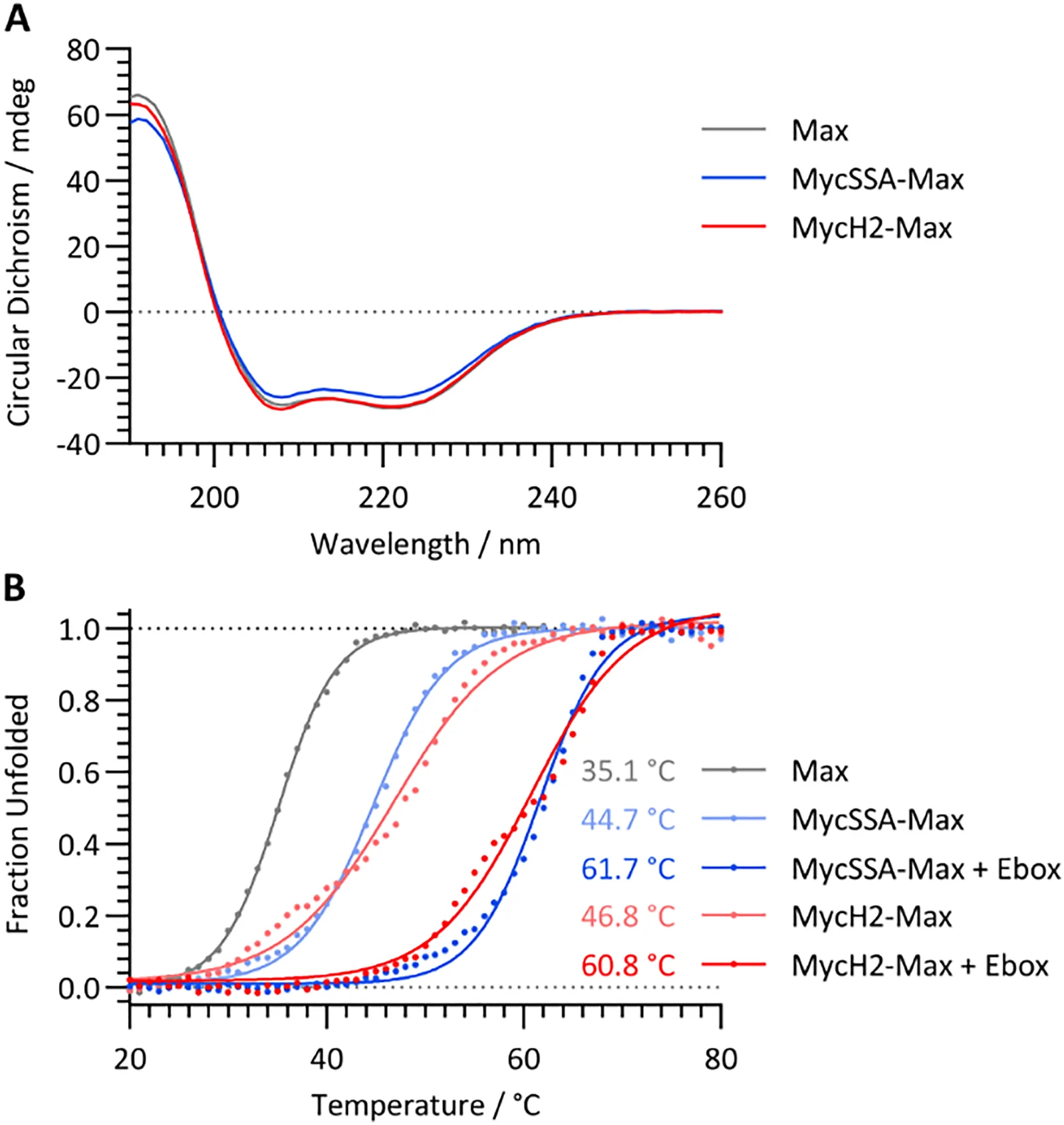
Stapling in helix two does not perturb c-Myc's native protein–protein and protein–DNA interactions. (A) Circular dichroism spectra of the unstapled MycSSA-Max and stapled MycH2-Max heterodimers as well as a Max homodimer. All three dimeric complexes adopt a typical α-helical coiled coil structure. (B) Thermal denaturation profiles of the Max homodimer, as well as MycSSA-Max and MycH2-Max, analysed alone and in the presence of E-box DNA. A ∼15 °C increase in *T*_m_ is seen for both c-Myc constructs, indicating the formation of a ternary complex.

Ellenbroek *et al.* demonstrated the importance of the helix-loop-helix domain for correctly orientating the basic domains for DNA engagement; covalently linked c-Myc basic domains did not bind E-box DNA *in vitro*.^14^ Within the HLH domain, helix one shows residual α-helical structure in isolation and contributes directly to DNA contact.^18,23^ In addition, phosphorylation of S373 within helix one is known to disrupt heterodimerisation, preventing ternary complex formation.^24^ Structural changes in helix one have therefore been shown to modulate DNA binding, and this region may require conformational flexibility for induced-fit DNA recognition. By contrast, helix two lies at the structural junction between the HLH motif and the leucine zipper domain. Although AlphaFold predicts a loop-helix-two salt bridge involving the lysine substituted in MycH2, experimental NMR studies indicate that the isolated c-Myc bHLHZip remains conformationally heterogeneous, with residual α-helical propensity concentrated within the basic region and helix one.^18,23^ The predicted interaction therefore need not be persistently populated in the isolated state, and its loss in MycH2 does not prevent formation of Max-dependent E-box complexes. The introduction of a constraint in helix two may therefore stabilise the relative orientation of the basic regions while preserving the dynamic requirements of DNA engagement. The positional specificity observed here is consistent with this structural logic: inducing helicity in helix two reinforces productive geometry, whereas stapling in helix one may restrict conformational flexibility, and constraints in the leucine zipper are distal to the DNA-binding interface.

This shows that the observed gain of function is not a generic consequence of intracellular bis-alkylation. Rather, it is a sequence- and geometry-specific effect arising from a precisely positioned constraint in helix two. Together, these data demonstrate that covalent modification can convert the c-Myc bHLHZip into a more ordered and functional state in a strictly position-dependent manner. This conformational reprogramming therefore reflects a local, constraint-induced shift in the accessible functional state.

To confirm that chemical constraint does not disrupt native c-Myc biology, the stapled constructs were also analysed in the presence of Max. All stapled c-Myc variants formed heterodimeric complexes with Max that adopted a canonical α-helical coiled-coil structure, comparable to that of the Max homodimer (Fig. 3A and Fig. S15). Thermal denaturation experiments showed that all Myc-Max heterodimers were more stable than the Max homodimer alone, and that addition of E-box DNA produced a further ∼15 °C stabilisation (Fig. 3B and Fig. S16, S17), consistent with productive ternary complex formation. Except for a modest destabilisation observed for the helix-one-stapled construct (MycH1), stapling had little effect on the stability or DNA-binding ability of the Myc-Max complex (Fig. S16 and Table S11). Therefore, stapling in helix two confers novel Max-independent DNA binding properties without preventing canonical Myc-Max or DNA-associated complex formation.

## Conclusions

The prevailing view is that c-Myc is functionally inert in isolation. Intrinsic disorder within the bHLHZip domain precludes both stable DNA binding and meaningful ligand engagement unless structure is imposed by its obligate partner, Max. Our findings demonstrate that this dependence has a substantial structural component. By enforcing α-helicity at a precisely positioned site within helix two, the c-Myc bHLHZip region can be converted into a more ordered, DNA-competent species capable of sequence-specific E-box recognition in the absence of Max. Importantly, this does not reflect non-specific stabilisation or recapitulation of the canonical Myc-Max architecture. Instead, it reveals a distinct, non-native conformational state that nevertheless supports sequence-specific DNA recognition. Intracellular crosslinking efficiency was comparable across all five constructs. However, the gain of function described above was only observed when stapling in helix two. This indicates that conformational reprogramming of IDPs is governed by structural topology rather than generic helix induction. More broadly, these results suggest that the apparent ‘undruggable’ nature of many intrinsically disordered proteins may arise not from an absence of functional or ligandable conformations, but from the difficulty of accessing such states independently of the binding partners that normally impose structure. By actively suppressing disorder without requiring partner occupancy, intracellular chemical constraint provides a route to expose latent folded states, enabling functional interrogation of disordered proteins and creating new entry points for ligand discovery.

## Methods

Stapling of c-Myc was undertaken during expression in *E. coli*. Briefly, BL21 (DE3) were transformed with a pET21a-His_6_-Myc expression vector and grown in 2xYT to an OD_600_ of 0.6. Expression was induced with 0.3 mM IPTG. mDBMB (1000x, in butanone) was added to the expression media to a final concentration of 400 µM over four additions at hourly intervals beginning 30 mins post-induction. The temperature was maintained at 37 °C until 30 mins prior to the final mDBMB addition, at which point it was reduced to 18 °C. The expression was incubated at 18 °C for a further 18 hours, harvested by centrifugation and purified under denaturing conditions.

EMSA experiments were performed using annealed fluorescent E-box or H-box oligonucleotides. Protein samples were incubated with DNA for 1 h at room temperature before analysis by agarose gel electrophoresis and fluorescence imaging. Circular dichroism spectra and thermal denaturation experiments were collected using refolded c-Myc and Max constructs in CD-compatible buffer. Because absolute protein concentrations could not be reliably determined by absorbance, relative concentrations of the c-Myc constructs were estimated using analytical size-exclusion chromatography peak area at 214 nm; the estimated concentrations used for CD analysis were approximately 10–20 µM, with a final DNA concentration of 4.4 µM. Full experimental conditions, including LC/MS acquisition and analysis parameters, are provided in the SI.

## Author contributions

R. A. J.: investigation, methodology, formal analysis, data curation, visualisation, writing – original draft, writing – review and editing. J. M. M.: conceptualisation, supervision, funding acquisition, project administration, methodology, formal analysis, writing – original draft, writing – review and editing.

## Conflicts of interest

JMM is CSO of Revolver Therapeutics and an advisor to Sapience Therapeutics. There are no other financial or commercial conflicts to declare.

## Supplementary Material

CB-007-D6CB00191B-s001

## Data Availability

The data supporting this article are available in the main text and the supplementary information (SI). Additional raw data are available from the corresponding author upon reasonable request. For additional materials and methods, quality control of all constructs by mass spectrometry and SDS-PAGE, as well as extended biophysical characterisation, please see the SI. See DOI: https://doi.org/10.1039/d6cb00191b.
