# Supplementary material for: Conformational reprogramming of c-Myc via intracellular cyclisation
Source: RSC Chem Biol. 2026 Sep 15;7(10):1991–6. doi: 10.1039/d6cb00191b (PMC13576623; doi:10.1039/d6cb00191b)
Supplement: Supplementary file 1 [file CB-007-D6CB00191B-s001.pdf]

Supplementary Information (SI) for RSC Chemical Biology.
This journal is © The Royal Society of Chemistry 2026

Supporting Information

Conformational reprogramming of c-Myc via intracellular cyclisation

Rachel A. Johnson[1] and Jody M. Mason[1,2,3]

[1]Department of Life Sciences, University of Bath, Bath, BA2 7AY, United Kingdom

[2]Revolver Therapeutics, Innovation Centre, University of Bath, BA2 7AY, United Kingdom

[3]To whom correspondence should be addressed: j.mason@bath.ac.uk

Extended Materials & Methods **2**

General growth conditions 2

Generation of double cysteine mutants by site directed mutagenesis 2

Expression and purification of c-Myc constructs 2

Cloning, expression and purification of Max D23-S107 bHLHZip construct 3

In vitro stapling of c-Myc constructs 3

Estimation of relative concentration by analytical size exclusion chromatography 4

Electrophoretic Mobility Shift Assays (EMSA) 4

Circular Dichroism (CD) 4

Mass Spectrometry 5

Supplemental Figures **6**

Optimisation of expression conditions 6

QC data for all expression constructs 7

Site-directed mutagenesis to prevent post-translational modification of the N-terminal glycine 10

In vitro stapling of leucine zipper c-Myc constructs 12

Normalisation of CD scans using area under the peak by size exclusion chromatography 13

Raw EMSA gel images 15

Additional CD analysis of the c-Myc constructs 19

Additional CD analysis of the Myc-Max heterodimers 22

CD analysis of the Max homodimer 24

Additional References **25**

Extended Materials & Methods

General growth conditions

E. coli cultures were grown using Lysogeny Broth (LB; 10 g L^{-1} tryptone, 5 g L^{-1} yeast extract, 5 g L^{-1} NaCl) or 2xYT (16 g L^{-1} tryptone, 5 g L^{-1} yeast extract, 5 g L^{-1} NaCl) at 37 °C with agitation (200 rpm). Solid media contained 15 g L^{-1} agar. Carbenicillin (100 µg mL^{-1}) was used in selective media.

Generation of double cysteine mutants by site directed mutagenesis

The pET21a-Myc constructs were amplified by whole plasmid PCR, using a 2x Q5 Master Mix (NEB) according to the manufacturer's protocol. The PCR product was purified, and blunt end ligation was achieved by incubating with T4 ligase (NEB), T4 polynucleotide kinase (ThermoFisher) and DpnI (ThermoFisher) in T4 ligation buffer at room temperature for 1 hour. Chemically competent DH10B *E. coli* were transformed with ligation product by heat shock, plated onto selective LB agar containing carbenicillin and incubated at 37 °C overnight. Successful introduction of the two cysteine mutations was confirmed by Sanger sequencing.

Expression and purification of c-Myc constructs

Chemically competent BL21 (DE3) *E. coli* were transformed with pET21a-Myc by heat shock, plated onto selective LB or 2xYT agar and incubated overnight. A single colony was used to inoculate an overnight culture in selective LB media. 500 mL 2xYT media was inoculated with 5 mL overnight culture and grown to OD ~0.6-0.8. Gene expression was induced with the addition of IPTG to a final concentration of 0.3 mM. mDBMB (1000x, in butanone) was added to the expression media over four additions at hourly intervals beginning 30 mins post-induction. The final mDBMB concentration was 400 µM in 0.1 % butanone. The temperature was maintained at 37 °C until 30 mins prior to the final mDBMB addition, at which point it was reduced to 18 °C. The expression was incubated at 18 °C for approximately 18 hours and harvested by centrifugation (5000 *x* g, 30 mins, 4 °C), the supernatant discarded and the pellets stored at -80 °C.

Cell pellets were thawed on ice and gently resuspended in lysis buffer (20 mM Tris pH 8.0, 500 mM NaCl, 1 mM DTT, 1% Triton X-100, 0.025 mg mL^{-1} DNase, 0.2 mg mL^{-1} Lysozyme, 1.25 mM $MgSO_4$, cOmplete EDTA-free Protease Inhibitor Cocktail) and incubated at room temperature with agitation for 1 hour. The suspension was then lysed by sonication (60 % amplitude, 10 mins, 10 secs on/ 10 sec off) and centrifuged (14,000 *x* g, 30 mins, 4 °C) to isolate the insoluble material.

For larger pellets, additional washes were carried out using a high salt (2 M NaCl) lysis buffer to remove contaminating DNA and the sample centrifuged as previously described.

Isolated insoluble material, including the c-Myc inclusion bodies, was resuspended by sonication in denaturing lysis buffer (20 mM Tris pH 8.0, 500 mM NaCl, 20 mM imidazole, 1 mM DTT, 8 M urea) and incubated overnight at 4 °C with agitation. If any insoluble material was still visible following this incubation, the volume of denaturing lysis buffer was increased, and the sample sonicated until complete dissolution was achieved. Denatured samples were centrifuged at 40,000 x g for 30 mins, the supernatant isolated and passed through a 0.45 µM MCE sterile filter.

2-3 mL HisPur Ni-NTA resin (ThermoFisher) was equilibrated in denaturing lysis buffer and incubated with the filtered denatured c-Myc supernatant for approximately 2 hrs at 4 °C with agitation. Following batch binding, nickel affinity chromatography was carried out manually using a gravity column. The resin was sequentially washed with 3-5 CVs of denaturing lysis buffer with increasing imidazole concentration (typically 60, 100, 300 and 500 mM).

Isolation of the inclusion bodies and the subsequent nickel-affinity chromatography was visualised by SDS-PAGE. Nickel-affinity elution fractions were flash frozen in liquid nitrogen and stored at -80 °C or refolded by dialysis. To refold the nickel-purified c-Myc constructs, aliquots were dialysed stepwise against dialysis buffer (20 mM Tris pH 8.0, 500 mM NaCl, 1 mM DTT) containing 4 M, 2 M, 1 M and finally 0 M urea. For subsequent analysis, the samples were dialysed further into a CD compatible buffer (20 mM KP_i pH 7.4, 150 mM KF). The degree of stapling by mDBMB was confirmed by mass spectrometry using an Agilent 6545 Q-ToF LC/MS (Agilent Technologies).

Cloning, expression and purification of Max D23-S107 bHLHZip construct

A codon optimised Max (D23-S107) bHLHZip sequence was amplified by PCR with an N-terminal His_6 tag using a 2x Q5 Master Mix (NEB) according to the manufacturer's protocol. Both the insert and pET21a vector were digested with NdeI (ThermoFisher) and XhoI (ThermoFisher) for approximately 3 hours. Alkaline phosphatase (NEB) was added to the vector digest for the final 30 mins. The Max D23-S107 bHLHZip insert digest was heat inactivated at 80 °C for 20 mins and purified. The vector digest was visualised by agarose gel electrophoresis, the band excised and purified by gel extraction. Purified vector and insert were ligated (approximate 1:3 molar ratio, total volume 10 µL) using T4 ligase (NEB) at 12 °C overnight. Chemically competent DH10B *E. coli* were transformed with ligation product by heat shock, plated onto selective LB agar containing carbenicillin and incubated at 37 °C overnight. Successful plasmid assembly was confirmed by Sanger sequencing.

Expression of His_6-Max (D23-S107) was carried out using the same method as the c-Myc bHLHZip constructs stated above. Once the large scale 500 mL cultures reached OD 0.6-0.8, expression was induced with the addition of IPTG to a final concentration of 0.3 mM, the expression temperature reduced to 18 °C and the cultures incubated overnight. Cultures were harvested by centrifugation at 5000 *x* g for 30 mins.

Cell pellets were resuspended in lysis buffer (20 mM Tris pH 8.0, 500 mM NaCl, 20 mM imidazole, 1 mM DTT, cOmplete EDTA-free Protease Inhibitor Cocktail) and lysed by sonication as previously described. Following sonication, the supernatant was isolated by centrifugation (40,000 x g, 30 mins) and passed through a 0.45 µM MCE sterile filter.

Nickel-affinity chromatography was carried out as described above for the c-Myc constructs and visualised by SDS-PAGE. The elution fraction(s) were concentrated using an Amicon Ultra centrifugal filter (Merck Millipore), centrifuged (15,000 *x* g, 10 mins) to pellet any insoluble material and purified further by size exclusion chromatography (SEC). 500 µL aliquots were loaded onto a 10/300 S75 Superdex Increase (Cytiva) column equilibrated in 20 mM KP_i pH 7.4, 150 mM KF. Fractions were analysed by SDS-PAGE, pooled, aliquoted and flash frozen for further analysis.

In vitro stapling of c-Myc constructs

In vitro stapling of c-Myc post-purification was investigated as an alternative means of obtaining the stapled leucine zipper constructs, MycZ1, MycZ2 and MycZ3. Briefly, the three constructs were expressed as described previously without the addition of mDBMB. Inclusion bodies containing unstapled material were isolated, purified by nickel-affinity purification and dialysed into 20 mM Tris pH 8.0, 150 mM NaCl. To 3 mL of dialysed sample, 750 µL ACN, 3.75 µL 100 mM TCEP pH 7.0 and 3.75 µL 50 mM mDBMB in ACN were added and incubated at room temperature for 6hrs. Having observed incomplete stapling by mass spectrometry, the reactions were supplemented with a further 1.88 µL 100mM TCEP and 1.88 µL 50 mM mDBMB and incubated at room temperature overnight. Precipitation was removed by centrifugation (15,000 *x*g, 10 mins) and the supernatant isolated. Degree of stapling was determined by mass spectrometry.

Estimation of relative concentration by analytical size exclusion chromatography

A 10/300 S75 Superdex Increase size exclusion column was equilibrated with 20 mM KP_i pH 7.4, 150 mM KF and 500 µL of each c-Myc and Max construct injected. Absorbance was monitored at 280 and 214 nm. As all the c-Myc constructs were length matched, the area under the peak at 214 nm was determined to enable normalisation of the raw CD data (see **Figure S8** and **Figure S9**). Although absolute protein concentrations could not be determined reliably by absorbance, the c-Myc samples used for CD analysis were estimated to be approximately 10–20 µM. SEC peak area at 214 nm was therefore used to ensure comparable relative protein concentrations between constructs.

Electrophoretic Mobility Shift Assays (EMSA)

Each of the oligonucleotide sequences used were diluted in nuclease free water to 1.25 µM. Equal volumes of [FAM]-E-box forward/E-box reverse and [FAM]-H-box/H-box oligonucleotides were combined separately, generating 5× stocks of each dsDNA at 625 nM. The two DNA samples were incubated at 90 °C for 2 mins, then allowed to cool on the bench. Please see **Table S8** for exact oligonucleotide sequences.

4 µL of protein was combined with 1 µL DNA and incubated at room temperature for 1 hour. 1 µL 6x TriTrack DNA Loading Dye (Thermo Fisher) was added to each sample and resolved by agarose gel electrophoresis (1% gel, 90 V, 20 mins). The gels were visualised by fluorescence using an Azure 400 Imager (Azure Biosystems).

Circular Dichroism (CD)

CD was done using a Chirascan V100 (Applied Photophysics). Each protein sample was centrifuged (15,000 *x* g, 10 mins) and 200 µL transferred to a 1 mm quartz cuvette. When analysing Myc-Max heterodimers, the respective samples were combined in equal volume prior to centrifugation. Blank corrected spectra were recorded between 190-280 nm at 20 °C. Thermal melts were typically recorded between 10-85 °C in 1 °C intervals with a tolerance of 0.1 °C and allowing 60 secs equilibration at each temperature.

For CD analysis done in the presence of DNA, 200 µL protein was supplemented with 8.8 µL 50 % glycerol and 1.1 µL 50 mM EDTA. Either 20 µL 50 µM dsDNA (E-box or H-box) or 20 µL nuclease free water was then added. This gave a final DNA concentration of approximately 4.4 µM. The samples were incubated on ice for approximately 1 hour before 200 µL was transferred to a chilled cuvette and incubated in the Chirascan at 5 °C for a further 15 mins. Scans were recorded between 190-280 nm at 5 °C and melts recorded as described above.

As it was not possible to definitively determine the molar concentration, raw ellipticity in mdeg was not converted to mean residue ellipticity (MRE). To compare the scans of each stapled c-Myc construct in isolation, the raw CD data was normalised relative to MycSSA using the area under the peak by SEC.

In order to determine the melting temperature (T_m) for each condition, the raw thermal denaturation data was first converted to fraction unfolded as described below. Upper and lower baselines were defined by linear regression, where T is the temperature in °C.

$$upper\ baseline\ (ub) = upper\ slope * T + upper\ y\text{-}intercept$$
$$lower\ baseline\ (lb) = lower\ slope * T + lower\ y\text{-}intercept$$

Fraction unfolded at each temperature was then calculated using the following equation, where θ_{222} is the ellipticity in mdeg at 222 nm.

$$Fraction\ Unfolded\ (FU) = \frac{\theta_{222} - lb}{ub - lb}$$

Fraction unfolded was then plotted against temperature and the melting temperature (T_m) determined by Boltzmann sigmoidal non-linear regression using GraphPad Prism.

Mass Spectrometry

Data was collected in positive ionisation mode using an Agilent 1260 Infinity II HPLC, Agilent 6545 QToF LC/MS, MassHunter Acquisition and analysed using Qualitative Analysis v7. All protein samples were analysed using an Agilent PLRP-S 300 Å 3.0 µm 2.1 x 50 mm column in H_2O/acetonitrile containing 0.1 % (v/v) formic acid at a flow rate of 0.2 mL min^{-1} and a column temperature of 60 °C.

The gas temperature was 350 °C, drying gas flow was 11 L min^{-1}, and the nebuliser gas was at 50 psi. The sheath gas temperature and flow were 380 °C and 11 L min^{-1} respectively. The VCap, Fragmentor and Skimmer voltages were 4500, 340 and 120 V respectively, and data was collected over an m/z range of 300-3200.

Supplemental Figures

Optimisation of expression conditions

The addition of 250 µM mDBMB to the media during expression of MycZ3 was found to be insufficient to fully staple the protein. This was resolved by increasing the final concentration to 400 µM. Staggered addition of the chemical crosslinker as described above was also required.

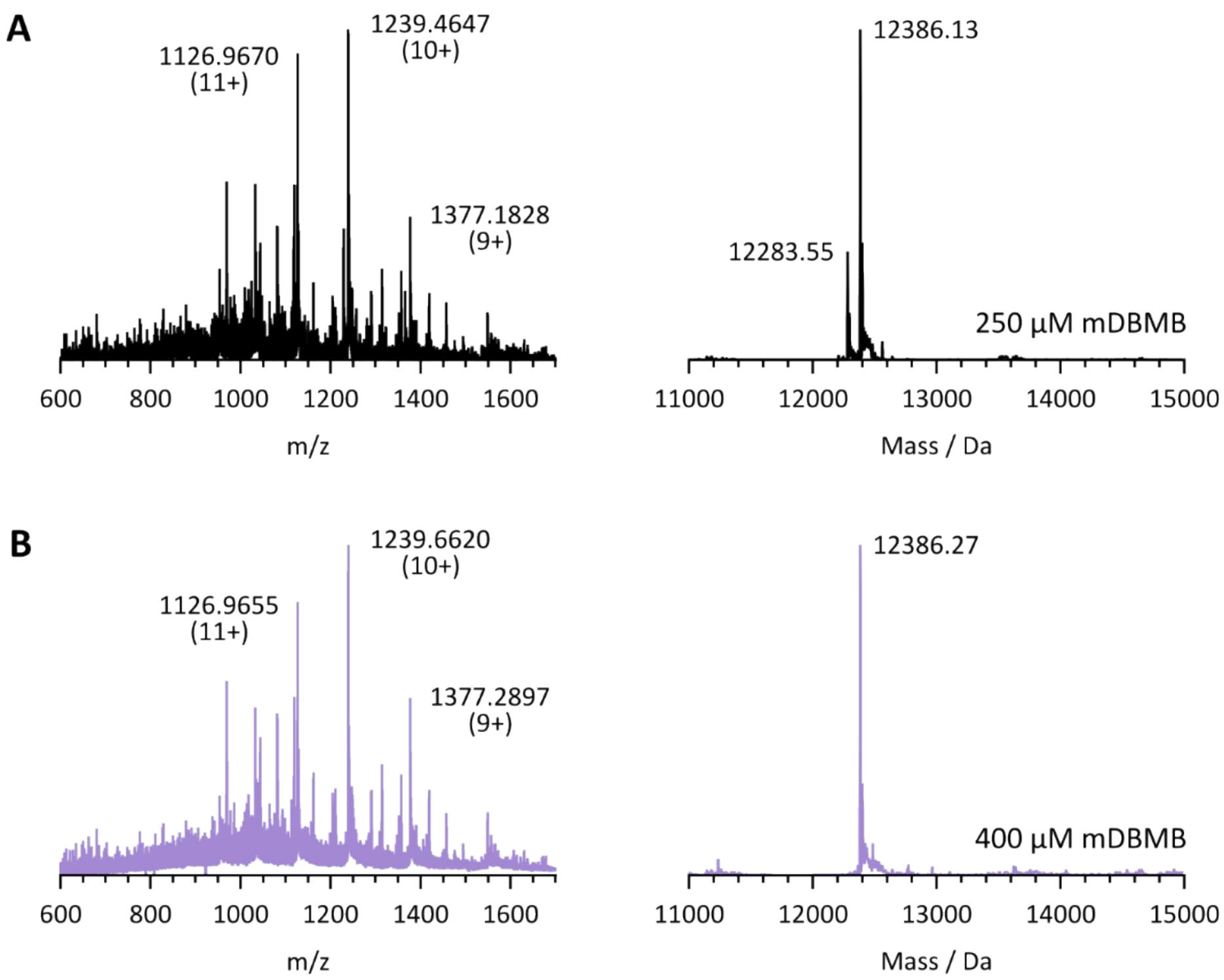

Figure S1: *Mass spectrometry analysis demonstrating the concentration dependent stapling of MycZ3 using **A**) 250 µM and **B**) 400 µM mDBMB. Unstapled MycZ3 was not observed at the higher mDBMB concentration. For each sample, the electrospray ionisation spectrum is given on the left and deconvoluted data on the right.*

Table S1: *Expected and observed molecular weights for stapled and unstapled MycZ3.*

Name	Expected Molecular Weight / Da	Observed Molecular Weight / Da
MycZ3	12284.044	12283.55
MycZ3-mDBMB	12386.180	12386.27

QC data for all expression constructs

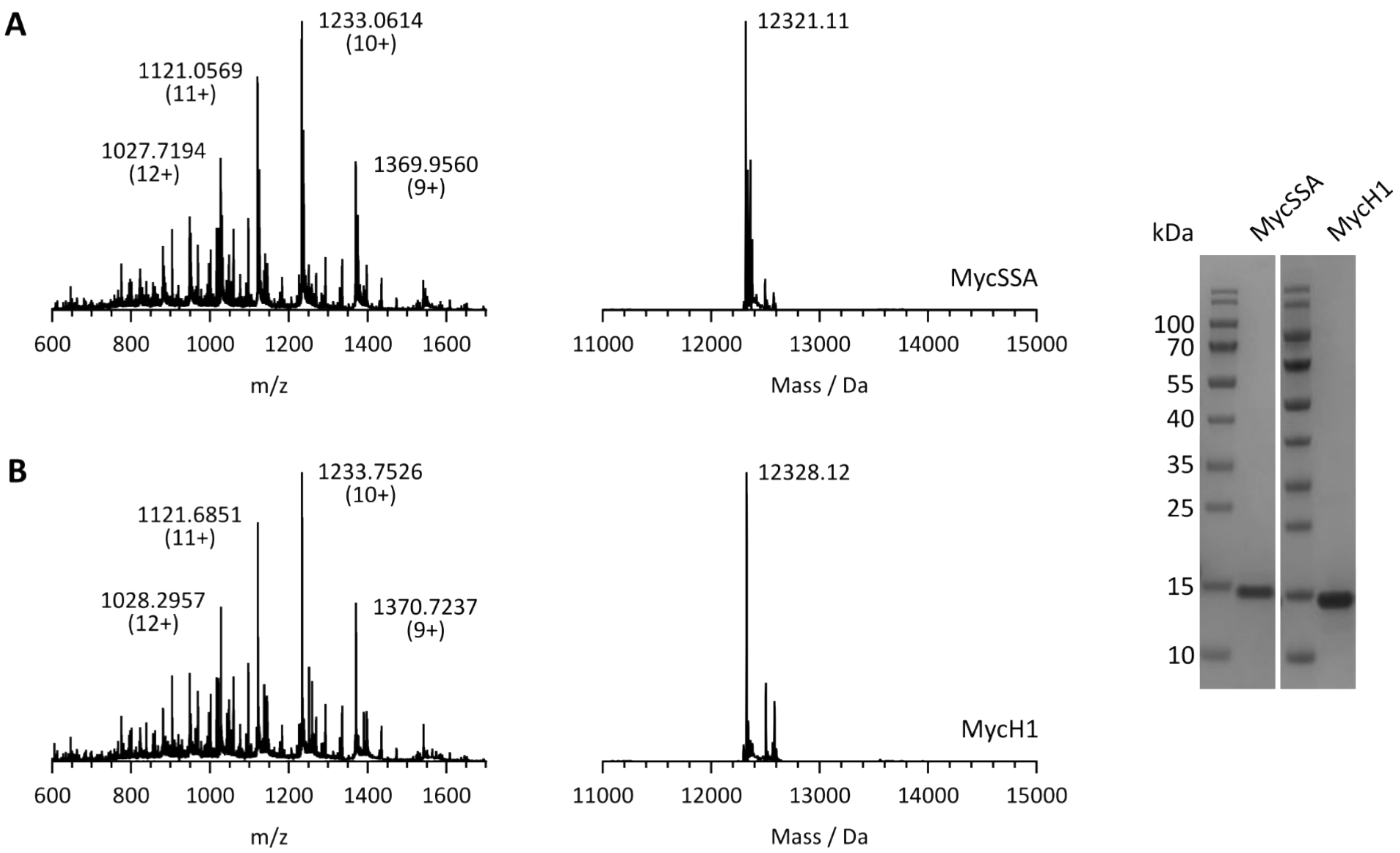

***Figure S2**: Mass spectrometry and SDS-PAGE QC data for MycSSA and MycH1. Electrospray ionisation spectra are given on the left and deconvoluted data on the right.*

***Table S2**: Expected and observed molecular weights for MycSSA and MycH1.*

		Molecular Weight / Da		
Construct	Sequence	Expected‡	(-Met)	Observed
MycSSA	MHHHHHHGSRSSDTEENVKRRTHNVLERQRRNELKRSFFALRDQIPELEN NEKAPKVVILKKATAYILSVQAEEQKLISEEDLLRKRREQLKHKLEQLRNSSA	12320.985	-	12321.11
MycH1	MGHHHHHHGSRSSDTEENVKRRTHNVLERQRRNELK**C**SFF**C**LRDQIPELE NNEKAPKVVILKKATAYILSVQAEEQKLISEEDLLRKRREQLKHKLEQLRNSS A	12459.183	12327.990	12328.12

‡ For all Myc constructs except MycSSA, the expected molecular weight includes the addition of mDBMB

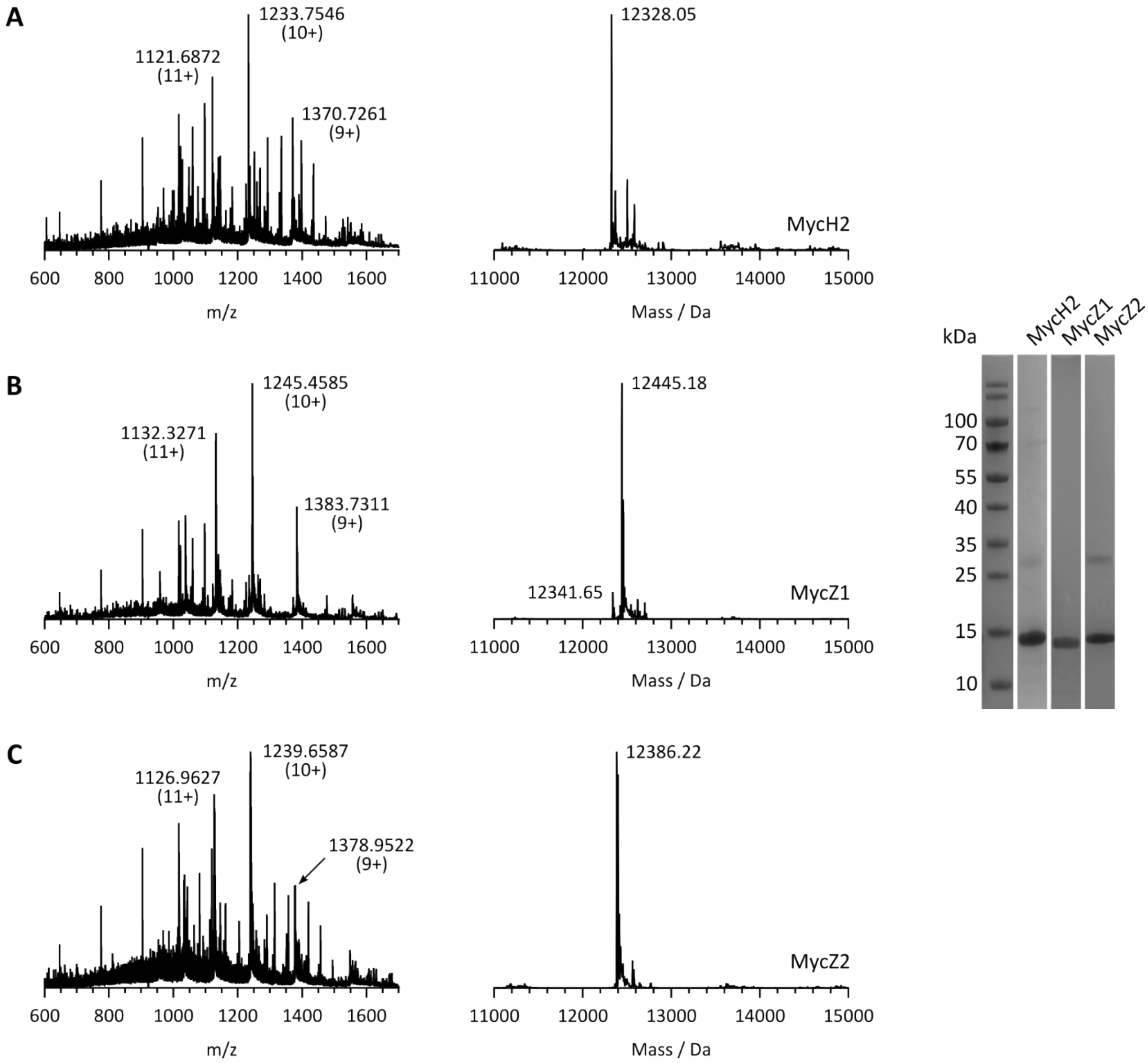

***Figure S3**: Mass spectrometry and SDS-PAGE QC data for MycH2, MycZ1 and MycZ2. Electrospray ionisation spectra are given on the left and deconvoluted data on the right.*

***Table S3**: Expected and observed molecular weights for MycH2, MycZ1 and MycZ2.*

		Molecular Weight / Da		
Construct	Sequence	Expected‡	(-Met)	Observed
MycH2	MGHHHHHHGSRSSDTEENVKRRTHNVLERQRRNELKRSFFALRDQIPELE<br>N<br>NEKAPKV**C**ILK**C**ATAYILSVQAEEQKLISEEDLLRKRREQLKHKLEQLRNSSA	12459.143	12327.950	12328.05
MycZ1	MHHHHHHGSRSSDTEENVKRRTHNVLERQRRNELKRSFFALRDQIPELEN<br>NEKAPKVVILKKATAYI**C**SVQ**C**EEQKLISEEDLLRKRREQLKHKLEQLRNSSA	12445.160	-	12445.18
MycZ2	MHHHHHHGSRSSDTEENVKRRTHNVLERQRRNELKRSFFALRDQIPELEN<br>NEKAPKVVILKKATAYILSVQAEEQKLISEE**C**LLR**C**RREQLKHKLEQLRNSSA	12386.136	-	12386.22

‡ *For all Myc constructs except MycSSA, the expected molecular weight includes the addition of mDBMB*

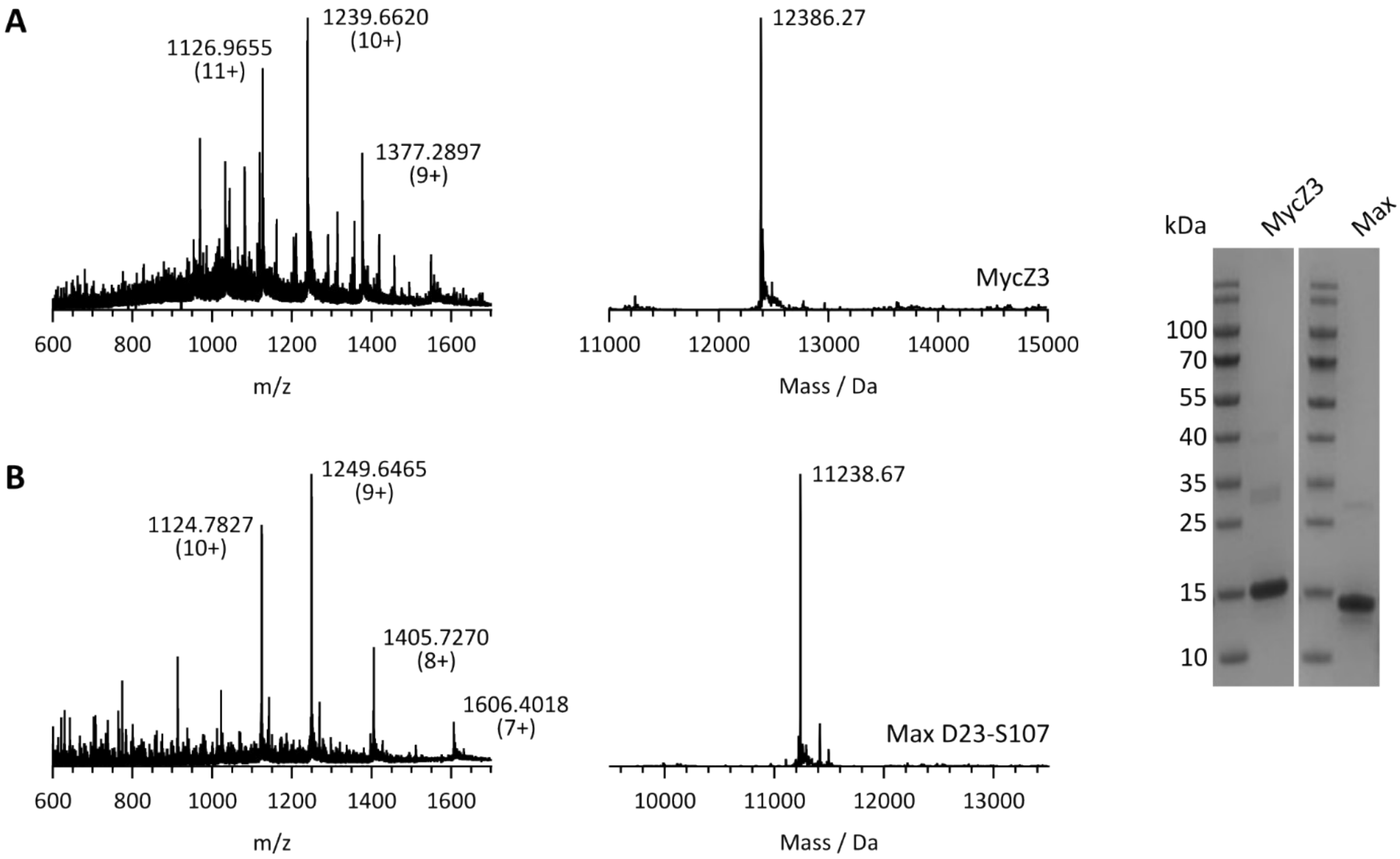

Figure S4*: Mass spectrometry and SDS-PAGE QC data for MycZ3 and Max D23-S107. Electrospray ionisation spectra are given on the left and deconvoluted data on the right.*

Table S4*: Expected and observed molecular weights for MycZ3 and Max D23-S107.*

		Molecular Weight / Da		
Construct	Sequence	Expected‡	(-Met)	Observed
MycZ3	MHHHHHHGSRSSDTEENVKRRTHNVLERQRRNELKRSFFALRDQIPELEN<br>NEKAPKVVILKKATAYILSVQAEEQKLISEEDLLRKRREQLKHKL**C**QLR**C**SSA	12386.180	-	12386.27
Max D23-S107	MHHHHHHASDKRAHHNALERKRRDHIKDSFHSLRDSVPSLQGEKASRAQ<br>ILDKATEYIQYMRRKNHTHQQDIDDLKRQNALLEQQVRALEKARS	11238.574	-	11238.67

‡ *For all Myc constructs except MycSSA, the expected molecular weight includes the addition of mDBMB*

Site-directed mutagenesis to prevent post-translational modification of the N-terminal glycine

Following mass spectrometry analysis of the stapled c-Myc constructs it became apparent that they were undergoing post-translational modification. The N-terminal methionine was being truncated and the glycine subsequently modified. This has been reported previously and is more likely when a glycine is present between the methionine and His_6 tag.[1, 2] Removal of this glycine by site directed mutagenesis was shown to prevent post-translational modification in this manner.

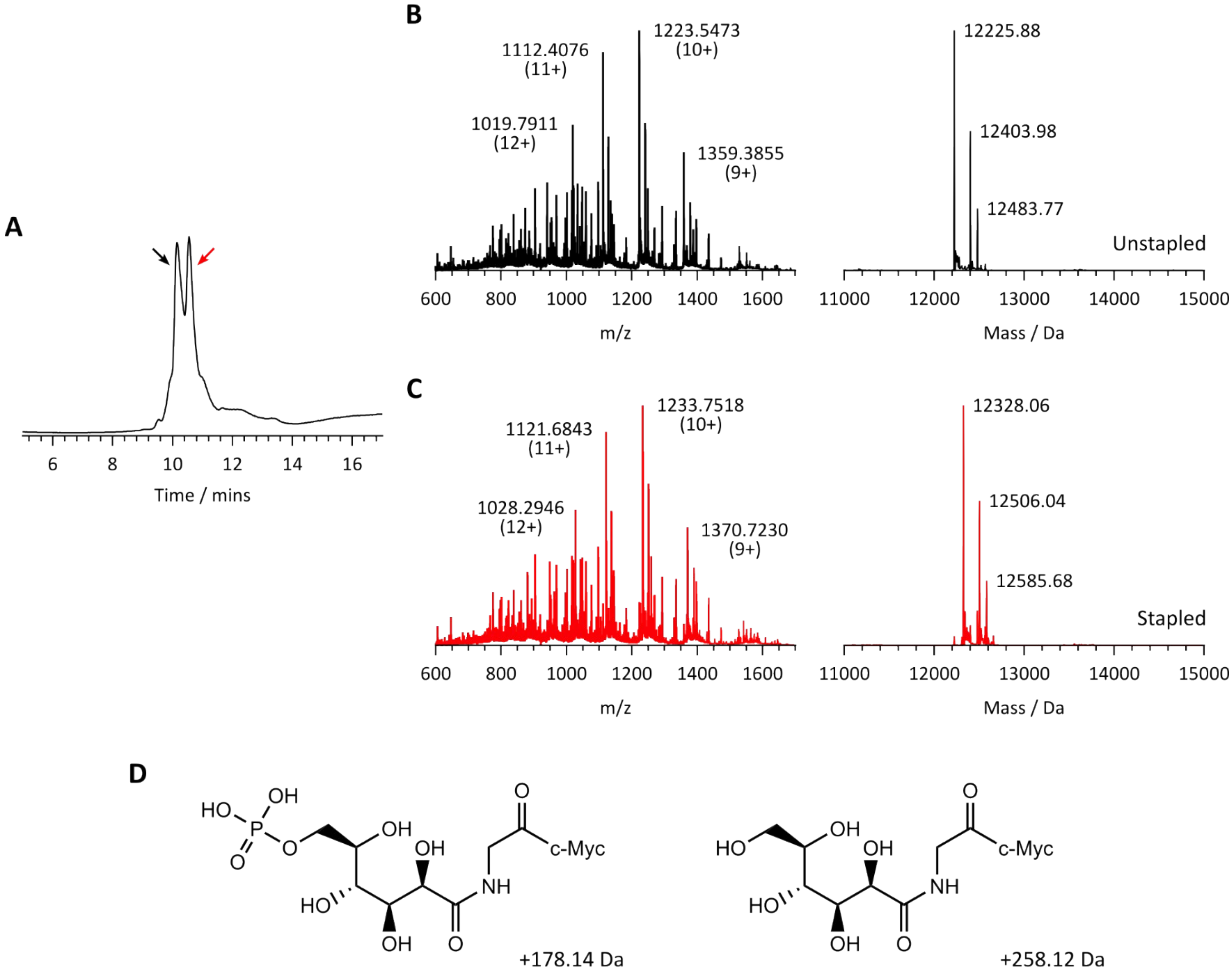

***Figure S5**: Mass spectrometry analysis of MycH2 showing the presence of two contaminants caused by post-translational modification. **A**) Two peaks are present in the TIC, corresponding to unstapled (black, **B**) and stapled (red, **C**) MycH2 respectively. Extracted ESI data (left) and deconvoluted (right) data demonstrates the presence of two contaminants at +178 Da and +258 Da caused by truncation of the N-terminal methionine and post-translational modification of the subsequent glycine. **D**) Structure of the two post-translational modifications observed.*

***Table S5**: Expected and observed molecular weights for MycH2.*

Name	Expected Molecular Weight / Da	Observed Molecular Weight / Da
MycH2	12357.007	-
MycH2 (-Met)	12225.814	12225.88
MycH2 (+mDBMB)	12459.143	-
MycH2 (-Met, +mDBMB)	12327.950	12328.06

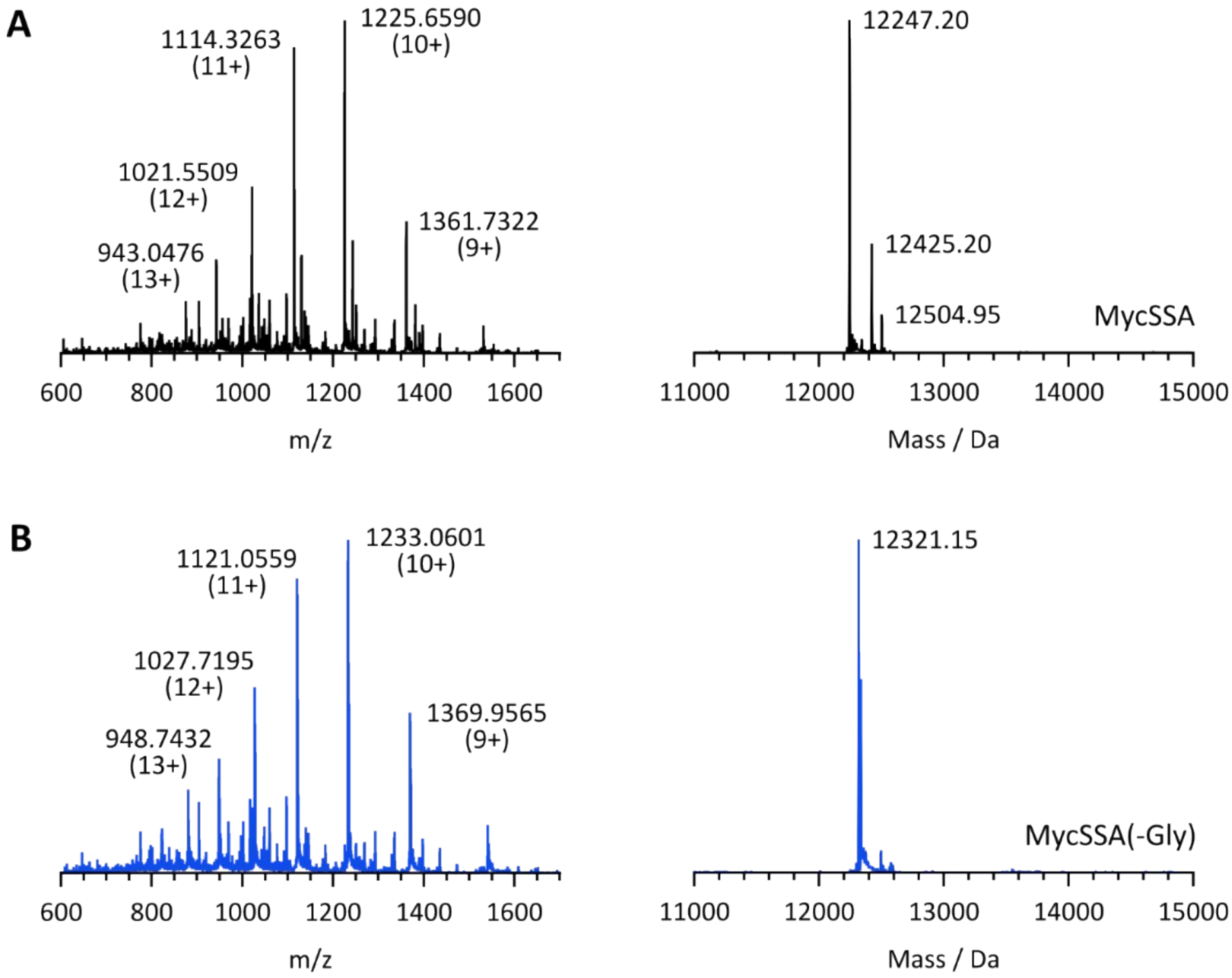

***Figure S6**: Mass spectrometry analysis of MycSSA before and after site-directed mutagenesis. Upon removal of the glycine between the N-terminal methionine and His_6 purification tag, methionine truncation and post-translational modification were no longer observed. This -Gly construct was used for all the analysis detailed herein but has been referred to as MycSSA throughout. As before, extracted ESI data is shown on the left and deconvoluted spectra on the right.*

***Table S6**: Protein sequences and molecular weights for MycSSA expression constructs, pre- and post-SDM.*

		Molecular Weight / Da		
Construct	Sequence	Expected	(-Met)	Observed
MycSSA	M**G**HHHHHHGSRSSDTEENVKRRTHNVLERQRRNELKRSFFALRDQIPELE N NEKAPKVVILKKATAYILSVQAEEQKLISEEDLLRKRREQLKHKLEQLRNSSA	12378.037	12246.844	12247.20
MycSSA (-Gly)	MHHHHHHGSRSSDTEENVKRRTHNVLERQRRNELKRSFFALRDQIPELEN NEKAPKVVILKKATAYILSVQAEEQKLISEEDLLRKRREQLKHKLEQLRNSSA	12320.985	12189.792	12321.15

***In vitro* stapling of leucine zipper c-Myc constructs**

In vitro stapling following expression and purification was explored as an alternative means of generating the stapled leucine zipper constructs. Minimal stapling was observed under aqueous conditions, and this was attributed to poor solubility of mDBMB in Tris buffer. Acetonitrile was added to the reaction buffer, and the degree of stapling was found to increase with increasing acetonitrile concentration. In addition, disulphide formation of the unstapled protein was observed, therefore necessitating the inclusion of a reducing agent. To prevent cross-reactivity with the free thiols in DTT, TCEP was used. However, it became apparent that at high concentrations, a mono-mDBMB-TCEP adduct can form.[3] A final TCEP concentration of 100 µM was found to be sufficient.

These conditions were taken forwards, as described in the Extended Materials & Methods above. However, all three samples also showed clear precipitation; this was particularly pronounced for MycZ2 and MycZ3. Only MycZ1 was amenable to further analysis and the data presented here are therefore limited. As demonstrated by the EMSA, MycZ1 did not bind E-box in the absence of Max but appeared to heterodimerise with Max with moderately reduced thermal stability in comparison to MycSSA. We therefore chose not to pursue this construct further.

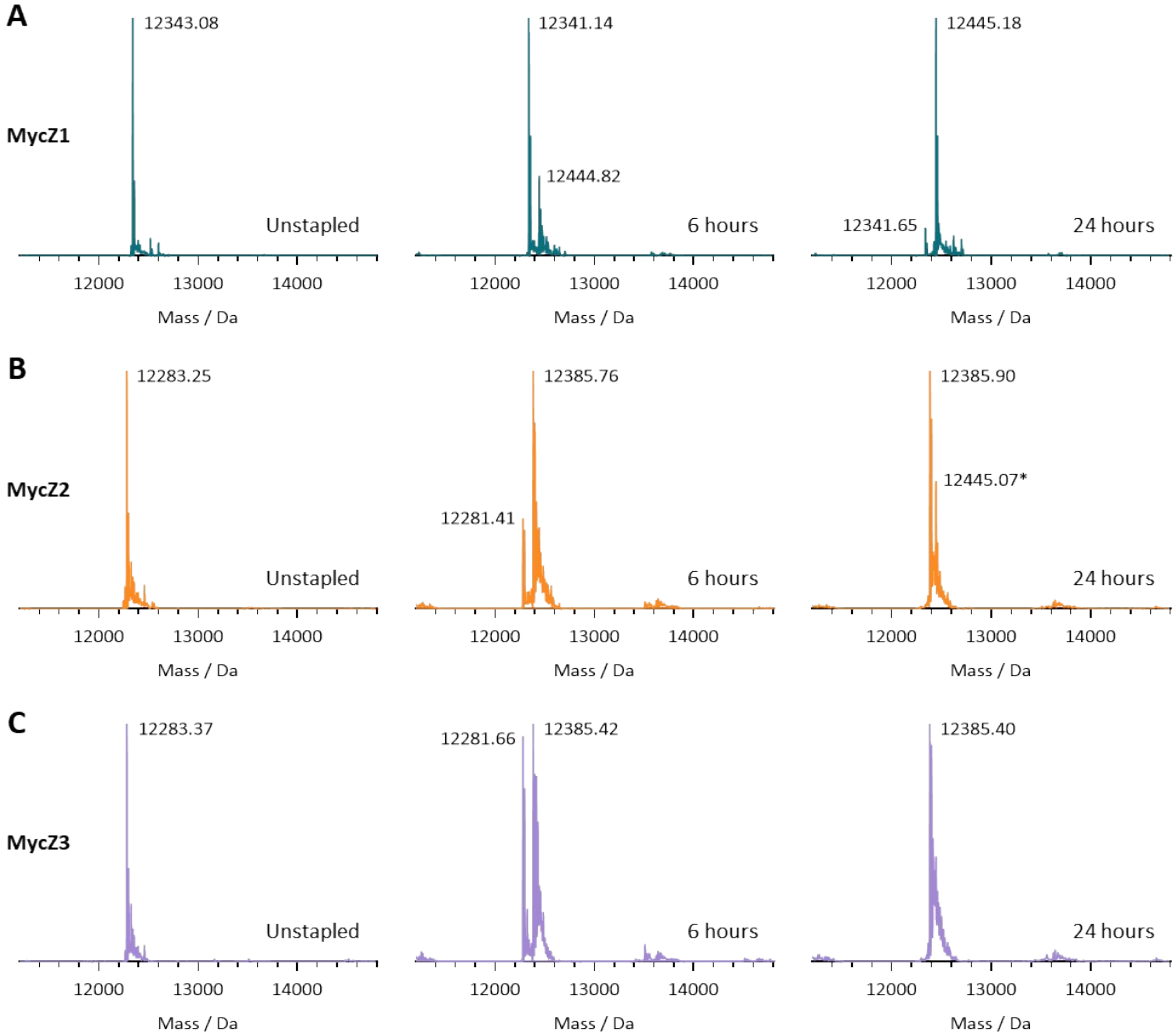

***Figure S7**: Mass spectrometry analysis of the leucine zipper constructs during in vitro stapling. Almost complete stapling was observed after 24 hours, with the exception of MycZ1. Significant precipitation was seen for MycZ2 and MycZ3, preventing further analysis. Disulphide formation is evident for the remaining unstapled sample after 6 hours. * indicates sample cross contamination from MycZ1 during MS analysis.*

Normalisation of CD scans using area under the peak by size exclusion chromatography

Due to c-Myc's limited solubility and the absence of sufficient aromatic residues, it was not possible to determine absolute concentration using absorbance-based methods. Instead, CD spectra were normalised relative to an unstapled control (MycSSA) using area under the peak integration by size-exclusion chromatography.

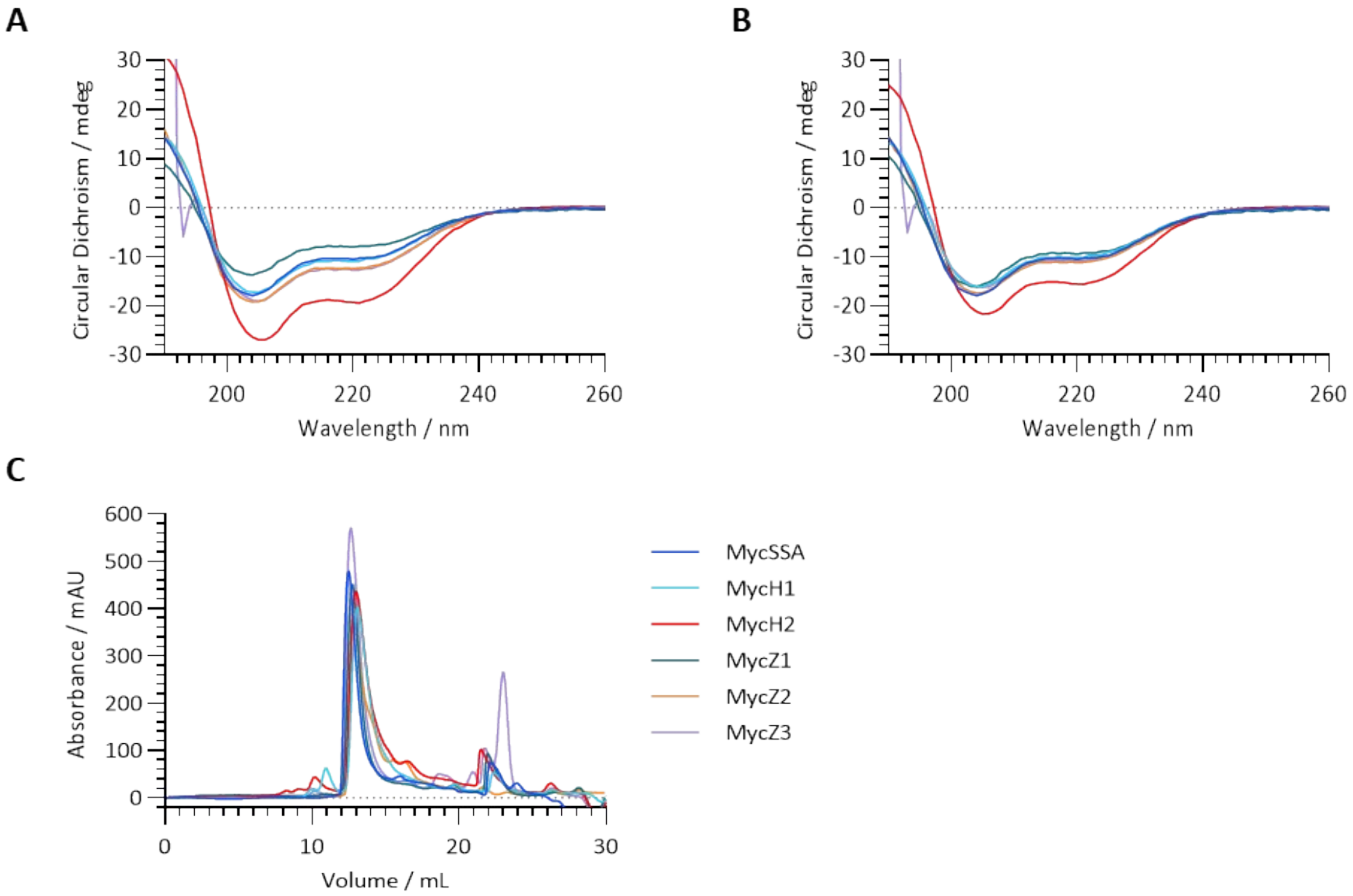

***Figure S8**: Raw (**A**) and normalised (**B**) CD spectra for each c-Myc construct, alongside the SEC chromatograms (**C**) used for normalisation. See **Table S7** for scale factors.*

***Table S7**: Scale factors used to normalise the raw CD data.*

Construct	Area under the peak	Scale factor
MycSSA (1/2)	654	1.00
MycSSA (2/2)	572	1.14
MycH1	702	0.93
MycH2 (1/2)	814	0.80
MycH2 (2/2)	600	1.09
MycZ1	556	1.18
MycZ2	724	0.90
MycZ3	764	0.86

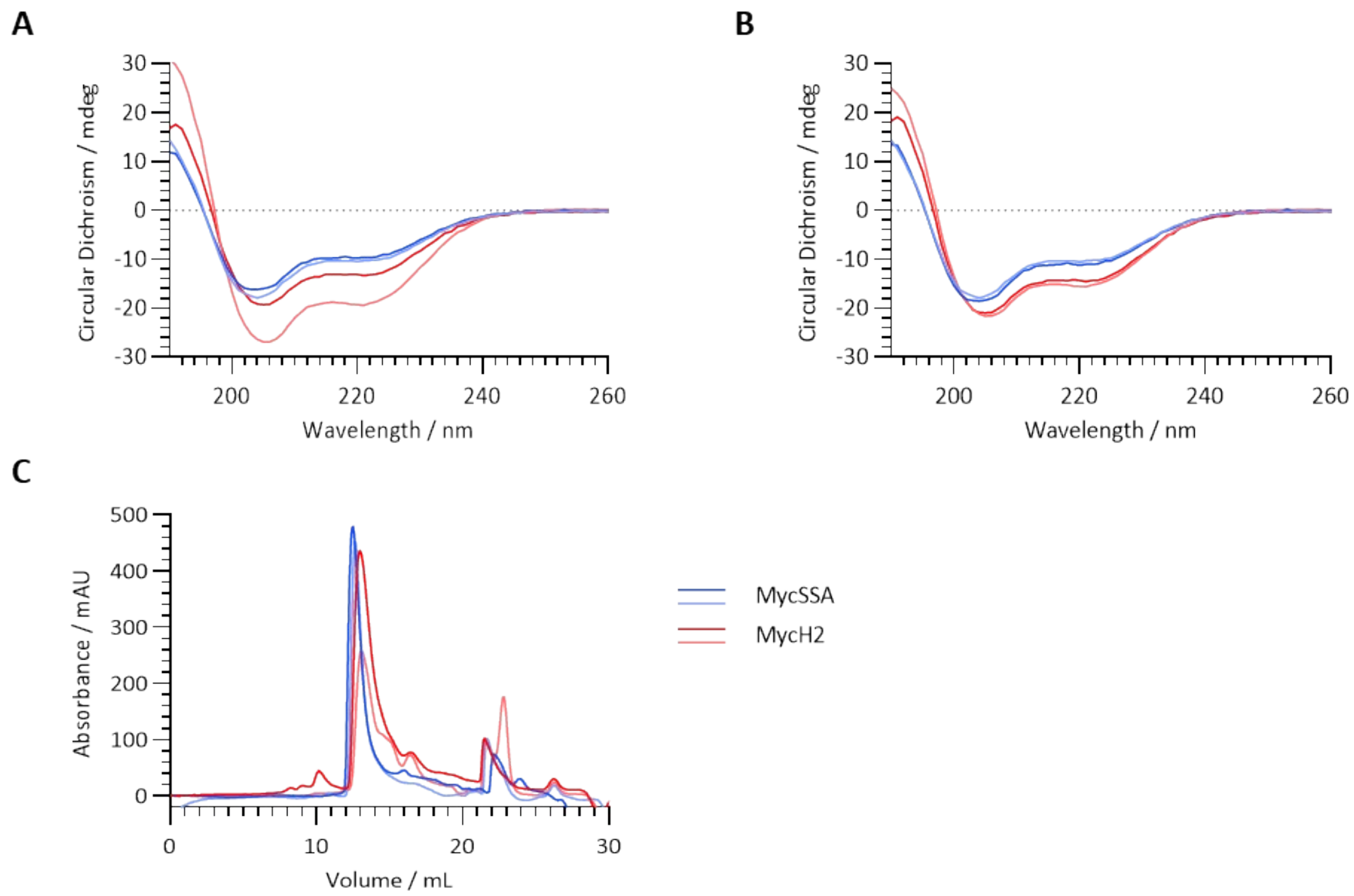

***Figure S9**: Raw (**A**) and normalised CD (**B**) spectra for two individual repeats of MycSSA and MycH2, alongside the SEC chromatograms (**C**) of each repeat used for normalisation. See **Table S7** for scale factors.*

Raw EMSA gel images

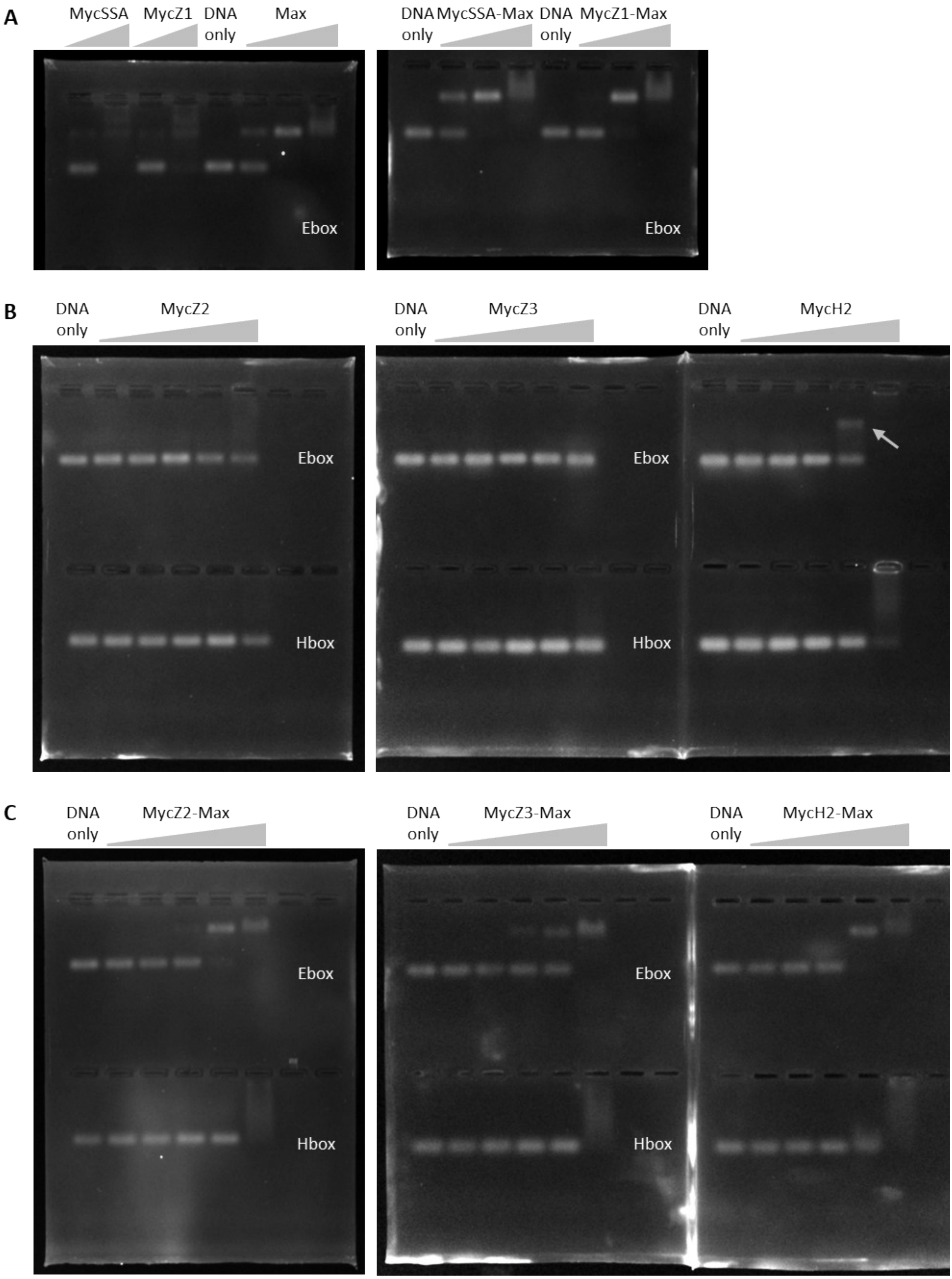

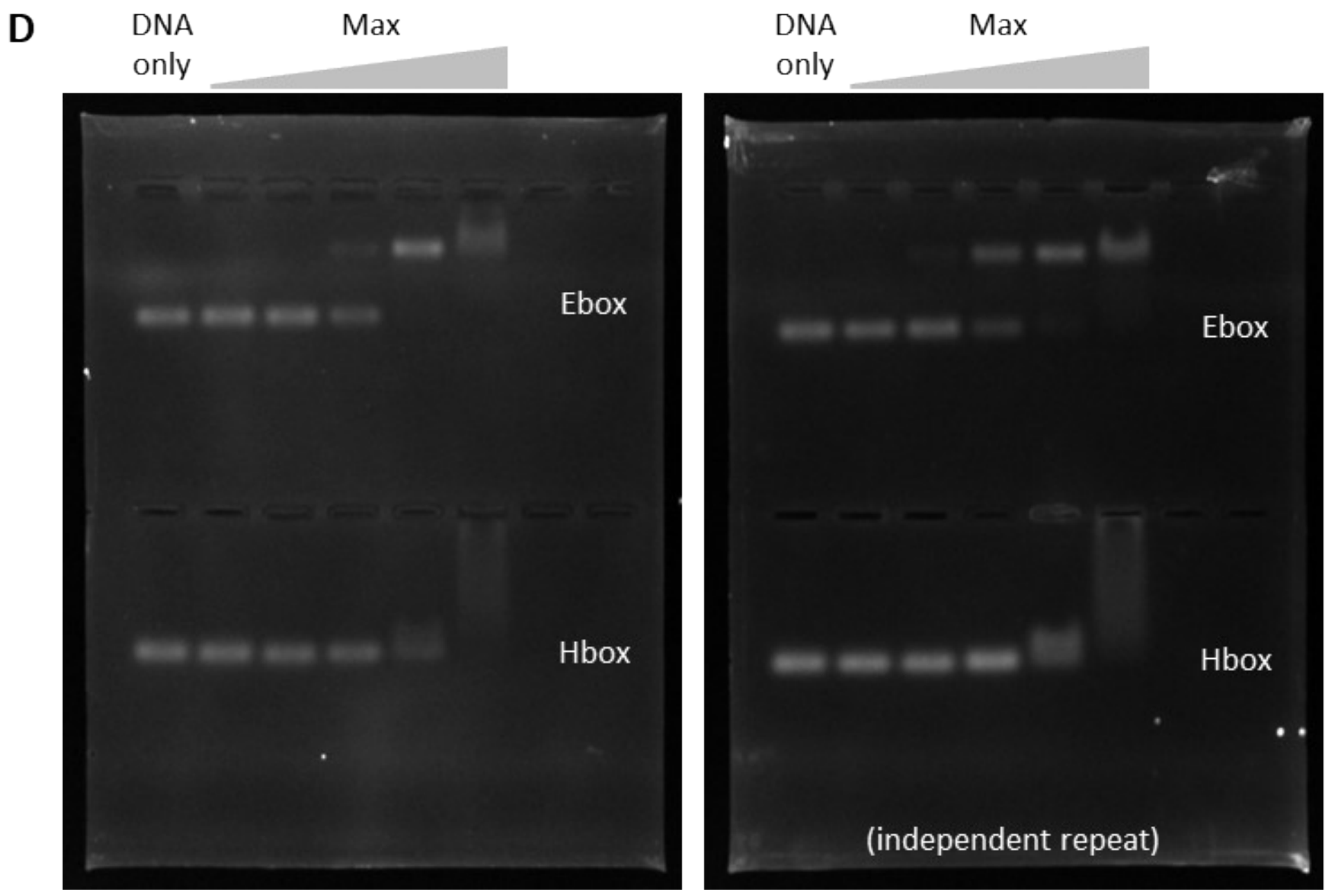

Figure S10*: Uncut EMSA gel images for MycSSA, MycH2, MycZ1-3 with and without Max, as well as the Max homodimer. The partial shift for MycH2 with E-box in the absence of Max is indicated by the arrow in panel B. Absolute protein concentrations could not be determined reliably; however, the same two-fold dilution series was used across all samples, with the rightmost lane for each sample containing undiluted protein. Relative concentrations were guided by SEC peak area at 214 nm as described above. Non-specific protein-DNA interactions in EMSA have been reported previously at high protein concentrations, which may explain the smearing of bands.[4]*

Table S8*: Oligonucleotide sequences used for EMSA analysis*

Name	Sequence (5' to 3')*
[FAM]-E-box fwd	[6FAM]-CAAGAC**CACGTG**GTCAAC
E-box rev	GTTGAC**CACGTG**GTCTTG
[FAM]-H-box	[6FAM]-TCTC**TAATTA**GAGA
H-box	TCTC**TAATTA**GAGA

**Binding regions are highlighted in bold.*

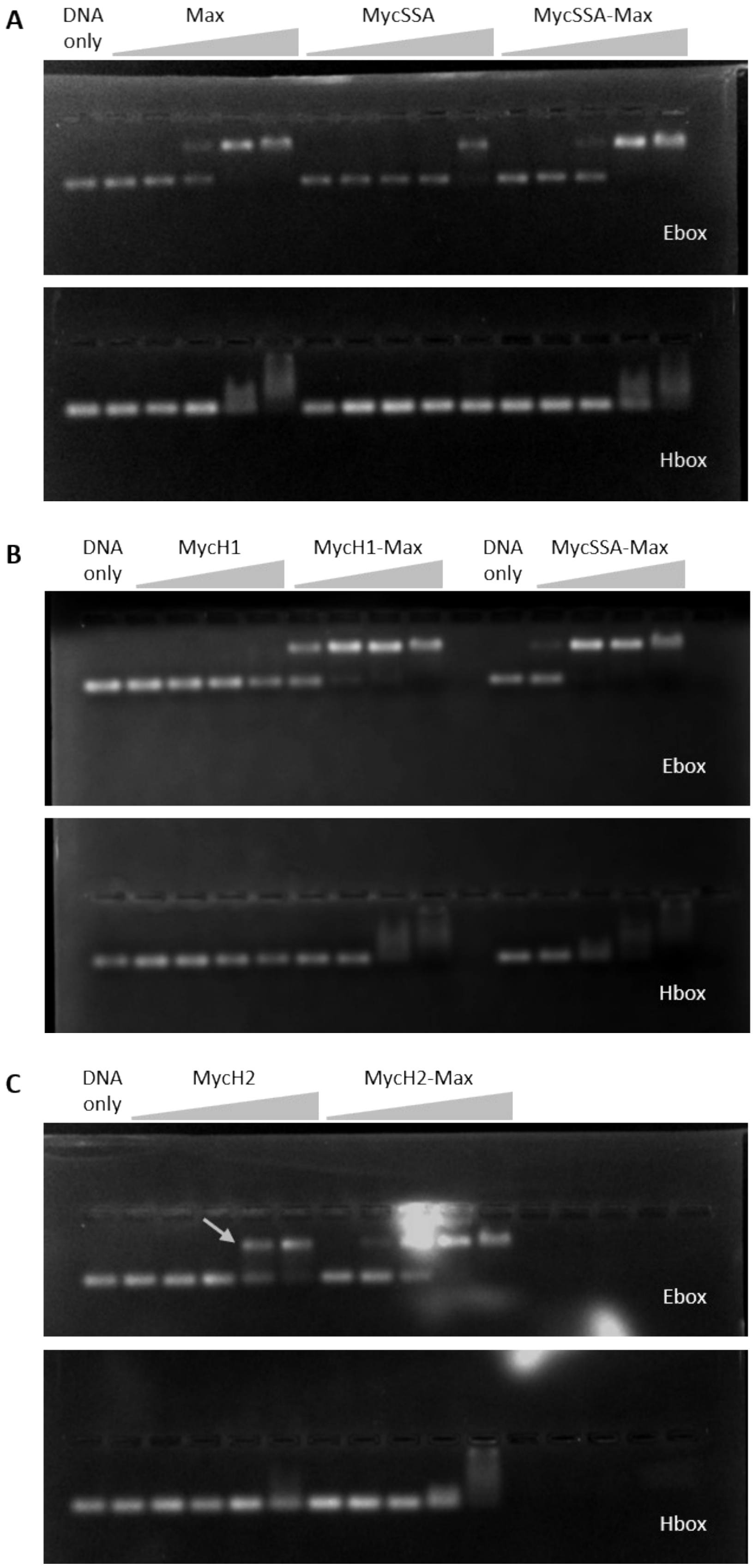
A
DNA only
Max
MycSSA
MycSSA-Max
Ebox
Hbox
B
DNA only
MycH1
MycH1-Max
DNA only
MycSSA-Max
Ebox
Hbox
C
DNA only
MycH2
MycH2-Max
Ebox
Hbox

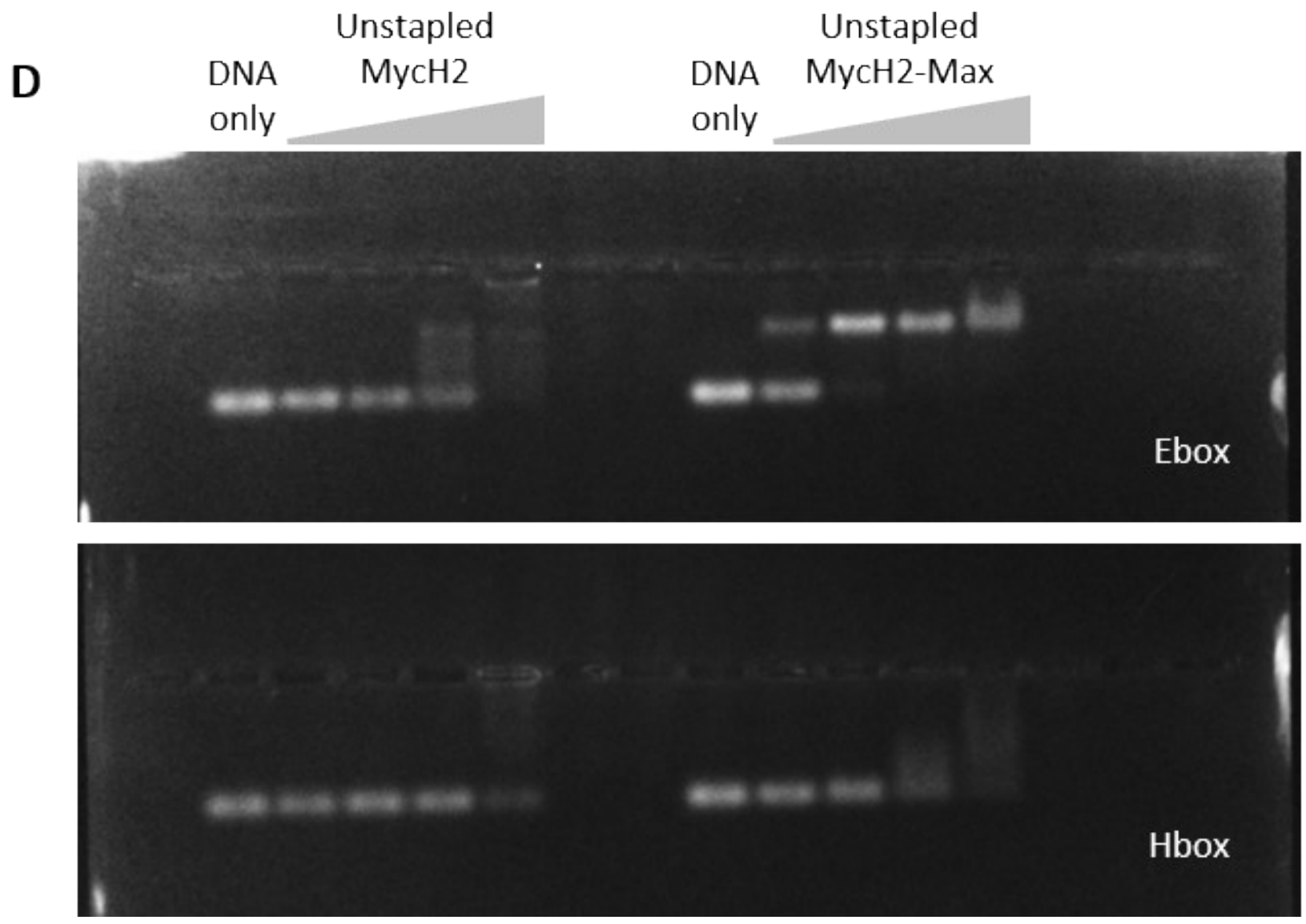

Figure S11*: Additional EMSA gel images for MycSSA, MycH1, stapled and unstapled MycH2, with and without Max. The partial shift for stapled MycH2 with E-box in the absence of Max is indicated by the arrow in panel C. Unstapled MycH2 does not produce a similar discrete Max-independent E-box shift, indicating that the gain of function requires mDBMB-mediated covalent cyclisation rather than cysteine substitution alone. As stated above, absolute protein concentrations could not be determined reliably; however, the same two-fold dilution series was used across all samples.*

Additional CD analysis of the c-Myc constructs

Thermal melt analysis of all c-Myc constructs showed a transition only upon addition of E-box DNA, indicating the interaction is sequence specific. When melted alone or in the presence of H-box DNA, a transition was not seen for any of the c-Myc constructs analysed (**Figure S12**). All of the analysis was done in the absence of Max. Notably, only MycH2 showed an increase in thermal stability (**Figure S13B**; **Table S9**). As shown in **Figure S14** and **Table S10**, MycZ2 and MycZ3 showed an increase in 222 nm/208 nm ratio upon the addition of E-box DNA. However, this did not translate to an increase in T_m. This suggests that although the leucine zipper regions of MycZ2 and MycZ3 became more helical upon addition of E-box DNA, this did not translate into increased thermal stability. Conversely, the introduction of a staple in helix one as in MycH1 appears to be detrimental to DNA binding, causing a reduction in the T_m (**Figure S13B**).

Wild-type c-Myc is widely reported to be unable to homodimerise under physiological conditions due to steric and electrostatic clashes in the leucine zipper. Therefore, the analysis shown below may partially reflect the assay conditions, with all components being used at low micromolar concentrations.

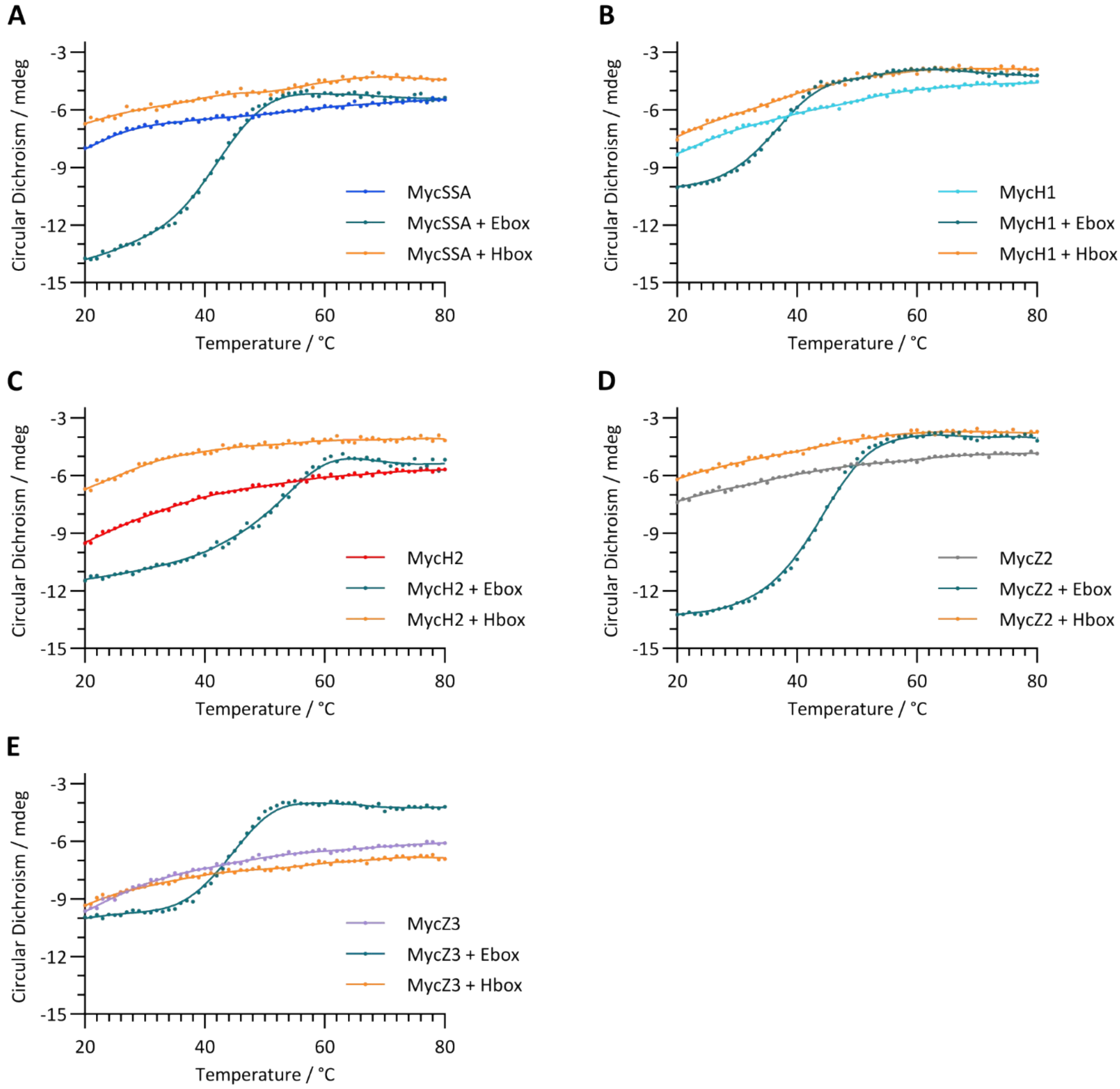

***Figure S12**: Thermal melts of all c-Myc constructs analysed alone, and in the presence of E-box or H-box DNA. A transition was only observed upon addition of E-box DNA, indicating the sequence specificity of this interaction.*

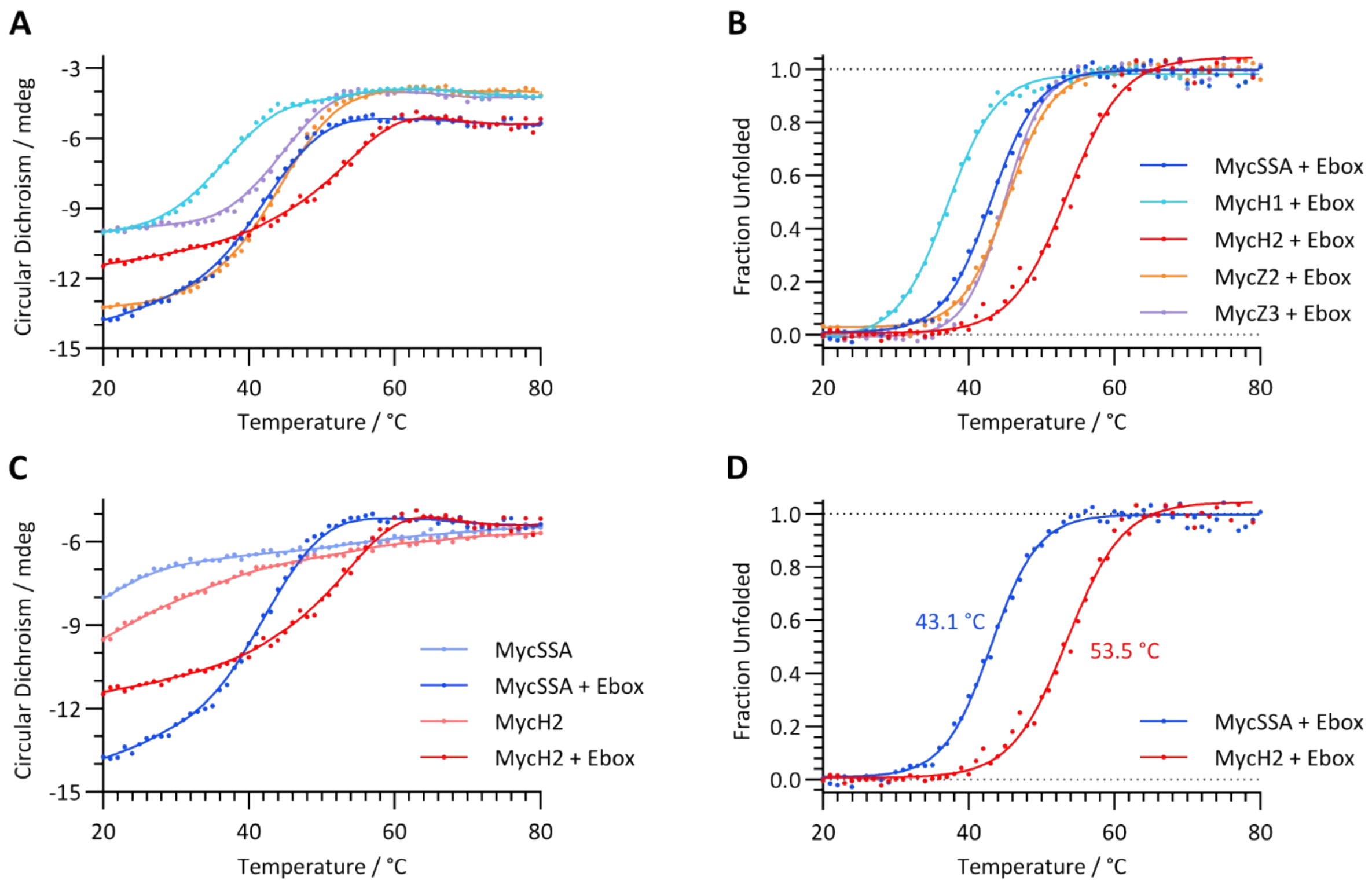

***Figure S13**: Comparison of raw and normalised thermal melt data for all c-Myc constructs following the addition of E-box DNA. **A**) Raw and **B**) normalised thermal melt data for all c-Myc + E-box samples, demonstrating a clear rightward shift only for MycH2 + E-box. This indicates that the increase in thermal stability is specific to stapling in helix two. **C**) Reproduction of Figure 2B comparing MycSSA and MycH2, with and without E-box DNA. **D**) The T_m values presented in the main text were generated using Boltzmann sigmoidal non-linear regression following normalisation of the data presented in panel C.*

***Table S9**: Melting temperatures for all c-Myc constructs following addition of E-box DNA.*

Construct	T_m / °C
MycSSA	43.1
MycH1	37.0
MycH2	53.5
MycZ2	45.3
MycZ3	44.9

A rightward shift in the minimum from ~205 nm to 208 nm was only seen upon addition of E-box DNA. For MycZ2 and MycZ3, this was accompanied by an increase in the 222 nm/208 nm ratio, which can be used as a measure of coiled coil character.[5] This suggests that stapling at these positions can induce helicity across the leucine zipper in DNA bound conformations.

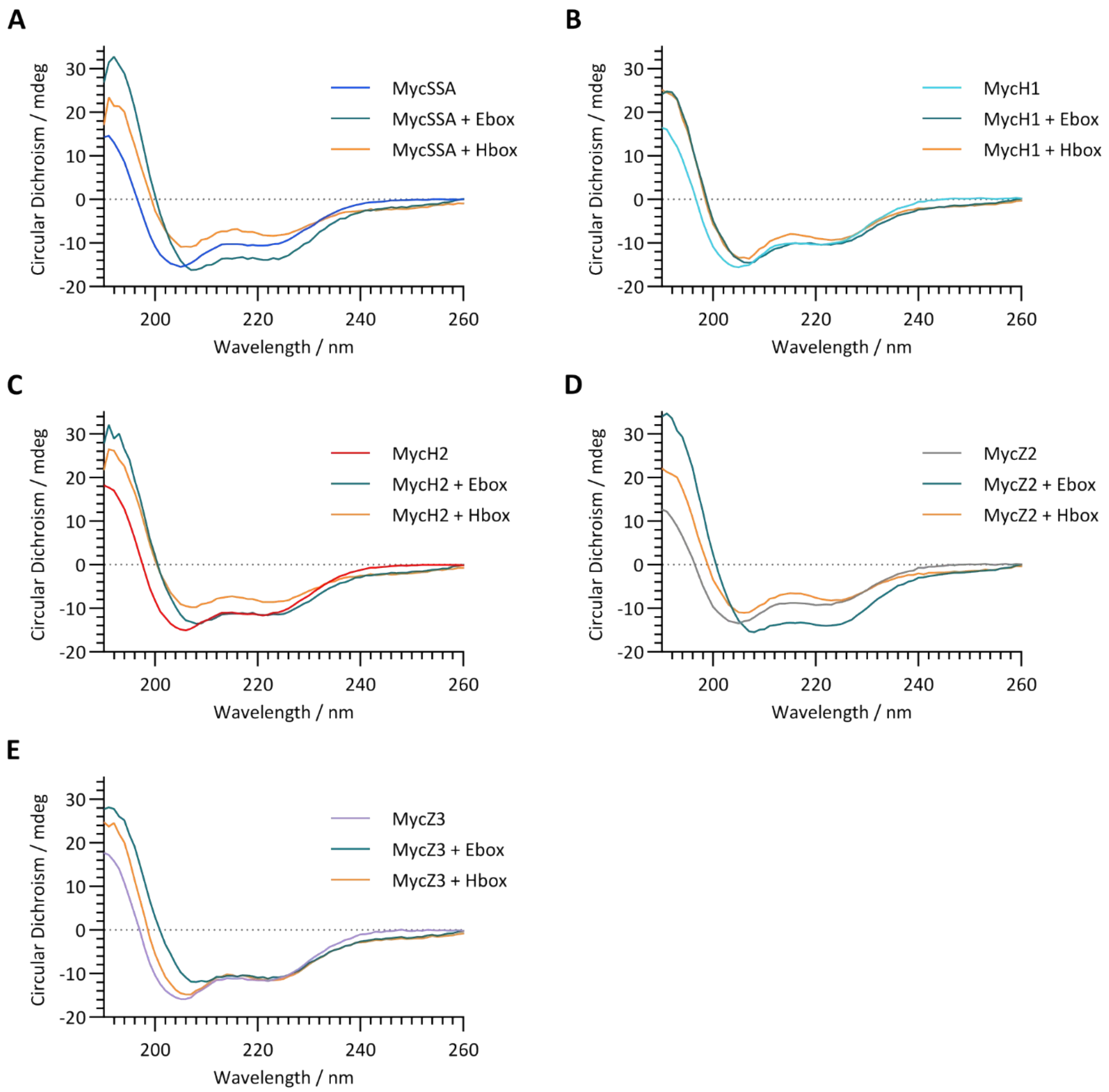

Figure S14: *CD scans of all c-Myc constructs analysed alone, and in the presence of E-box or H-box DNA. For all constructs, a rightward shift in the minimum from ~205 nm to 208 nm was seen only upon addition of E-box DNA.*

Table S10: *Ratio of circular dichroism at 222 and 208 nm for each c-Myc construct*

Construct	CD Ratio at 222 nm/208 nm		
	No DNA	+ E-box	+ H-box
MycSSA	0.76	0.86	0.79
MycH1	0.73	0.72	0.73
MycH2	0.82	0.86	0.88
MycZ2	0.76	0.90	0.79
MycZ3	0.81	0.94	0.83

Additional CD analysis of the Myc-Max heterodimers

All c-Myc constructs adopt a coiled coil conformation in the presence of Max. An increase in helicity (**Figure S15**) and thermal stability (**Figure S16**) was seen upon addition of E-box DNA, demonstrating that stapling does not prevent the formation of a ternary complex between Myc, Max and E-box DNA. The addition of a staple in helix one (MycH1) appears to be detrimental to E-box binding in the absence of Max (**Figure S13B**) and this is somewhat reflected here. The MycH1-Max-E-box complex has the lowest T_m of all those analysed (**Table S11**).

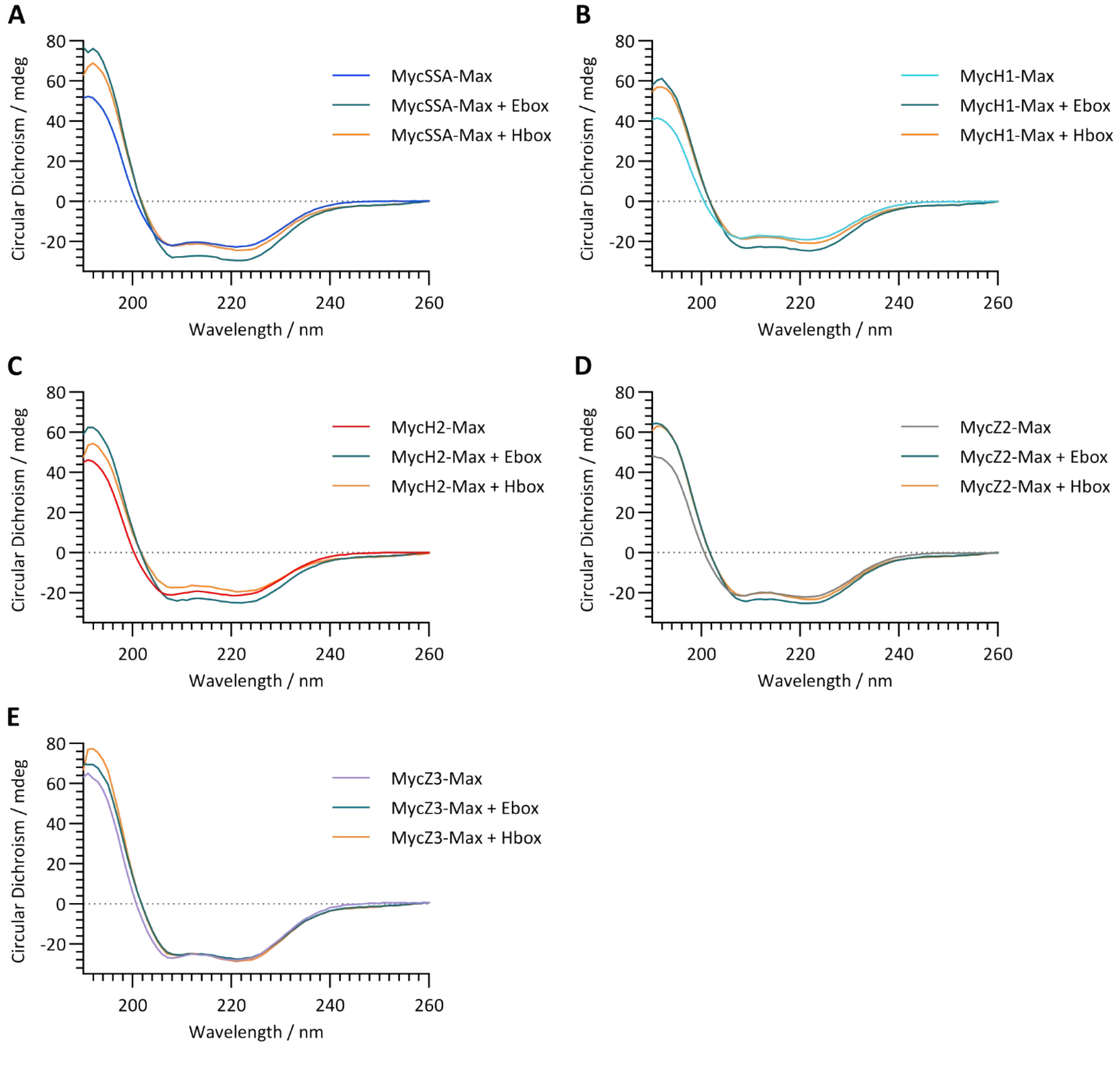

Figure S15*: CD scans of all Myc-Max heterodimers analysed alone, and in the presence of E-box or H-box DNA. Typically, an increase in helicity is seen upon addition of E-box DNA.*

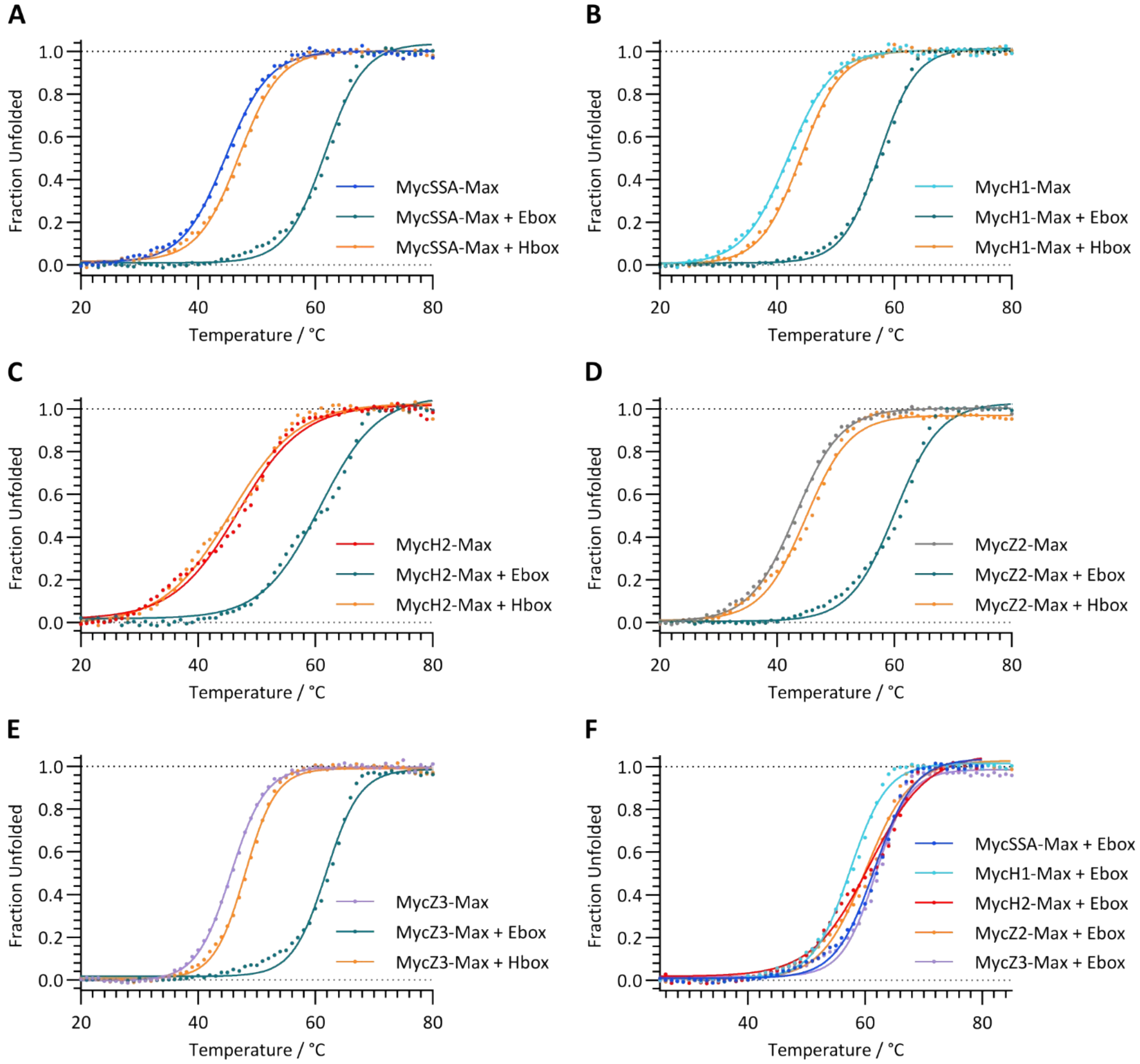

Figure S16*: Thermal melts of all Myc-Max heterodimers analysed alone, and in the presence of E-box or H-box DNA. For all constructs, a ~15 °C ΔT_m was only observed upon addition of E-box DNA (green), demonstrating the sequence specificity of this interaction. All T_m values are summarised in* ***Table S11*** *below.*

Table S11*: Melting temperatures observed for all Myc-Max heterodimers, alone or in the presence of E-box or H-box DNA.*

Construct	Melting Temperature (T_m) / °C		
	No DNA	+ E-box	+ H-box
MycSSA-Max	44.7	61.7	47.0
MycH1-Max	41.9	57.4	44.0
MycH2-Max	46.8	60.8	45.5
MycZ2-Max	43.1	60.1	44.9
MycZ3-Max	45.6	61.8	48.0
Max-Max	35.1	54.6	39.6

CD analysis of the Max homodimer

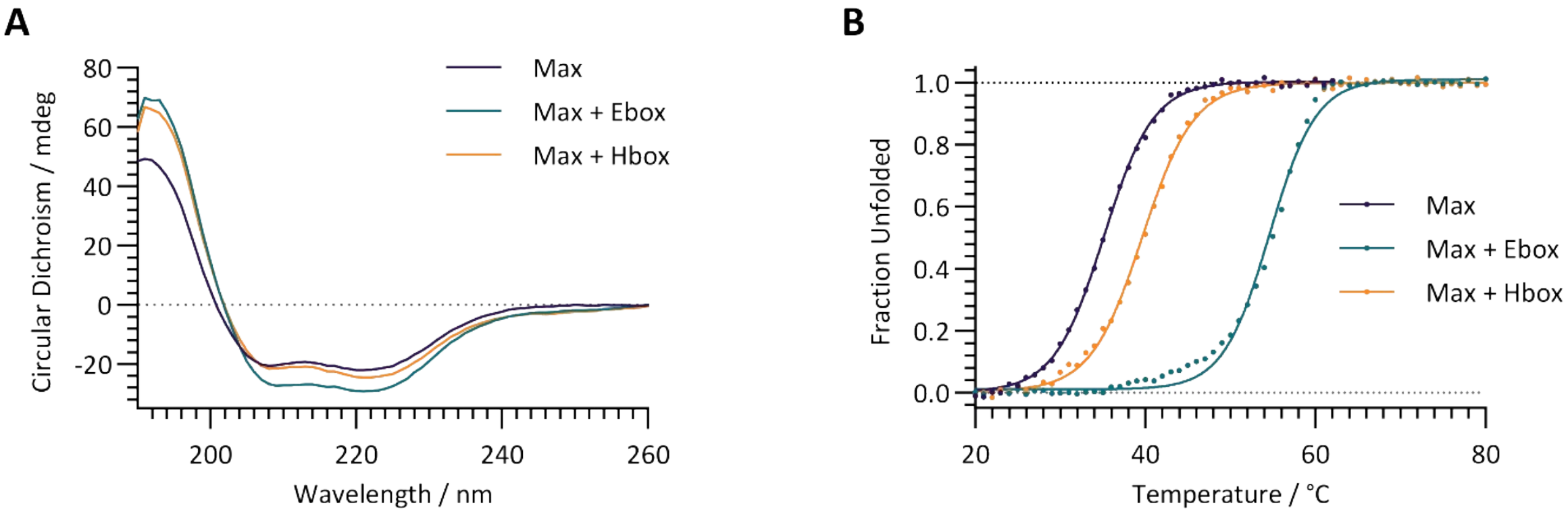

***Figure S17**: CD scans and thermal melts of the Max homodimer analysed alone, and in complex with E-box or H-box DNA. A comparable ~15 °C ΔT_m was observed upon addition of E-box DNA, as seen for the Myc-Max heterodimers shown in **Figure S16** above. Exact T_m values are stated in **Table S11**.*

To facilitate direct comparison of the Max-independent and Max-dependent E-box-bound states, the corresponding CD spectra, thermal denaturation profiles and 222/208 nm ratios are compared directly in Figure S18 and Table S12.

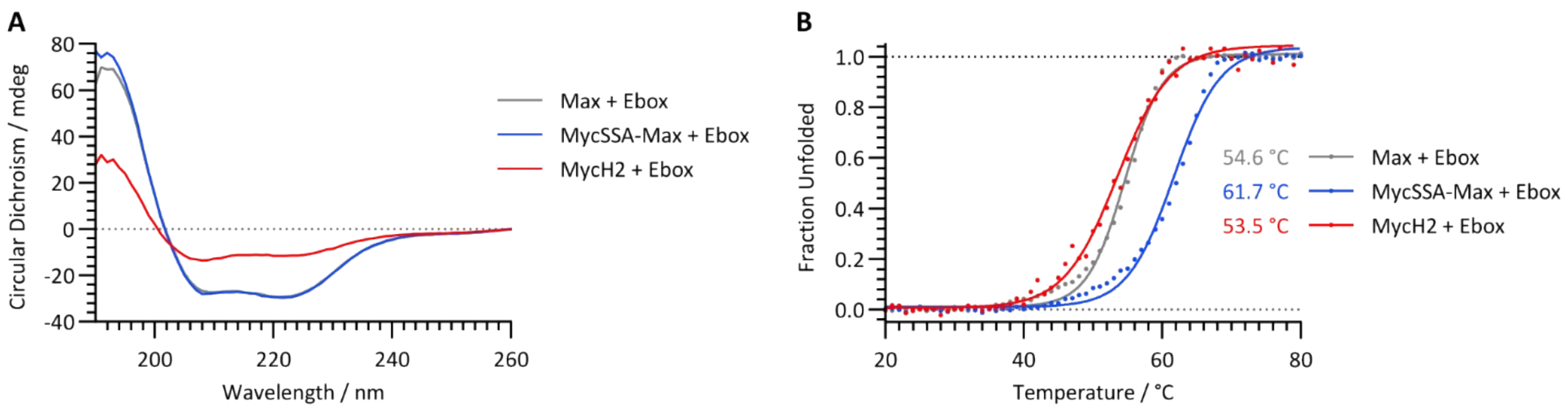

***Figure S18:** Direct comparison of Max-dependent and Max-independent E-box-bound states. **A**) Circular dichroism spectra of the Max homodimer + E-box, MycSSA-Max heterodimer + E-box, and stapled MycH2 + E-box. The MycH2 + E-box complex exhibits a CD profile distinct from the more strongly α-helical Max-containing complexes, consistent with formation of a non-canonical Max-independent DNA-bound state. **B**) Corresponding normalised thermal denaturation profiles. Note that T_m values and 222 nm/208 nm ratios are summarised in **Table S12** below.*

***Table S12:** Comparison of 222 nm/208 nm ratio and melting temperatures for Max-dependent and Max-independent E-box bound states.*

Complex	222 nm / 208 nm Ratio	Melting Temperature (T_m) / °C
Max + Ebox	1.09	54.6
MycSSA-Max + Ebox	1.05	61.7
MycH2 + Ebox	0.86	53.5

Additional References

(1) Geoghegan, K.; Dixon, H.; Rosner, P.; Hoth, L.; Lanzetti, A.; Borzilleri, K.; Marr, E.; Pezzullo, L.; Martin, L.; LeMotte, P.; et al. Spontaneous α-N-6-phosphogluconoylation of a "His tag" in Escherichia coli: The cause of extra mass of 258 or 178 Da in fusion proteins. *Analytical Biochemistry* **1999**, *267* (1), 169-184. DOI: 10.1006/abio.1998.2990.

(2) Martos-Maldonado, M.; Hjuler, C.; Sorensen, K.; Thygesen, M.; Rasmussen, J.; Villadsen, K.; Midtgaard, S.; Kol, S.; Schoffelen, S.; Jensen, K. Selective N-terminal acylation of peptides and proteins with a Gly-His tag sequence. *Nature Communications* **2018**, *9*. DOI: 10.1038/s41467-018-05695-3.

(3) Peraro, L.; Siegert, T.; Kritzer, J. Conformational Restriction of Peptides Using Dithiol Bis-Alkylation. In *Peptide, Protein and Enzyme Design*, **2016**; Vol. 580, pp 303-332.

(4) Ellenbroek, B.; Kahler, J.; Arella, D.; Lin, C.; Jespers, W.; Zueger, E.; Drukker, M.; Pomplun, S. Development of DuoMYC: a synthetic cell penetrant miniprotein that efficiently inhibits the oncogenic transcription factor MYC. *Angewandte Chemie-International Edition* **2025**, *64* (4). DOI: 10.1002/anie.202416082.

(5) Lau, S. Y. M.; Taneja, A. K.; Hodges, R. S. Synthesis of a model protein of defined secondary and quaternary structure. *Journal of Biological Chemistry* **1984**, *259* (21), 13253-13261. DOI: 10.1016/S0021-9258(18)90686-1.
